# Cold environment is associated with worse outcomes in ischemic stroke patients and the underlying gut microbial mechanism

**DOI:** 10.1186/s12866-026-05013-8

**Published:** 2026-04-01

**Authors:** Chanjuan Wei, Xiao Zhou, Xiaoshuang Xia, Wenjun Feng, Lin Wang, Xin Li

**Affiliations:** 1 Department of Neurology, The Second Hospital of Tianjin Medical University, No.23, PingJiang Road, Tianjin, 300211 China; 2 Department of Geriatrics, The Second Hospital of Tianjin Medical University, No.23, PingJiang Road, Tianjin, 300211 China; 3 Tianjin Interdisciplinary Innovation Centre for Health and Meteorology, Tianjin, China

**Keywords:** Gut microbiota, Ischemic stroke, Cold environment, Prognosis, Microbiota‒gut‒brain axis

## Abstract

**Background:**

Increasing evidence suggests that cold environment is a potential risk factor for acute ischemic stroke (AIS) and is associated with poor prognosis, however, its underlying mechanisms remain unclear. Since gut dysbiosis and AIS are causally related and cold environment can induce changes in the gut microbiota, we wondered whether cold-season-related gut dysbiosis aggravates stroke progression.

**Methods:**

In total, 101 patients with AIS were enrolled and divided into two groups: cold-season onset ischemic stroke (CIS) and non-cold-season onset ischemic stroke (NCIS). Gut microbiota composition was analyzed using 16 S rRNA gene sequencing, and signature taxa were identified via linear discriminant analysis effect size (LEfSe). Correlations between key microbial taxa and clinical parameters were assessed using Spearman rank analysis. To evaluate the potential causal role of gut microbiota in cold season stroke, fecal microbiota transplantation (FMT) was performed in mice, followed by middle cerebral artery occlusion (MCAO).

**Results:**

The composition of gut microbiota in the CIS group significantly differed from the NCIS group. The characteristic microbiota of the CIS group was distinguished by an elevated relative abundance of *Escherichia-Shigella* and *Enterococcus*, coupled with a decreased percentage of *Blautia*,* Eubacterium_hallii_group*,* Subdoligranulum*,* Dorea*,* Faecalibacterium*,* Ruminococcus*, and *Collinsella*. Furthermore, *Escherichia-Shigella*, *Enterococcus*, *Blautia*, *Eubacterium_hallii_group* and *Faecalibacterium* showed predictive value for 3-month poor prognosis in patients. Compared with mice inoculated with the NCIS gut microbiota, mice inoculated with the CIS gut microbiota showed more severe brain damage, impaired intestinal barrier function, and higher levels of inflammatory factors after the stroke model was established.

**Conclusions:**

Our study indicates that cold-season-related gut dysbiosis may be linked to stroke severity and poor prognosis in AIS patients, suggesting that modulation of gut microbiota could represent a potential avenue for therapeutic intervention.

**Supplementary Information:**

The online version contains supplementary material available at 10.1186/s12866-026-05013-8.

## Introduction

Climate change has been increasingly identified as one of the major causes of public health hazards on a global scale. Notably, global warming does not equate to a reduction in the threat posed by cold to public health; conversely, sustained increases in global temperatures may heighten susceptibility to risks associated with cold [1]. Among these, acute ischemic stroke (AIS) has garnered particular attention. Recent studies have demonstrated that cold season environment not only elevates the risk of AIS onset but also associated with more severe neurological deficits and poorer clinical outcomes [2, 3]. To date, the precise mechanisms by which cold environment contributes to the onset and progression of ischemic stroke remain unclear; however, several potential pathways have been reported, including cold-induced vasoconstriction, increased blood viscosity, heightened inflammatory responses, platelet aggregation, and enhanced sympathetic nervous system activity [4, 5]. This has led to growing concerns regarding the influence of environment temperature on the onset and development of stroke.

Increasing evidence support the crucial role of gut microbiota and its metabolites in the occurrence and development of stroke [6]. Patients poststroke exhibit an imbalance in the gut microbiota, characterized by the enrichment of potentially pathogenic microbes and the depletion of beneficial bacteria. A pivotal study revealed that ischemic stroke rapidly induces gut dysbiosis; conversely, disordered gut microbiota in turn exacerbates brain infarction [7]. Research has indicated that both dietary and environmental factors have greater influences on the makeup of the microbial community than genetic factors do [8]. Temperature has been recognized as an important environmental factor influencing both the composition and function of the gut microbiota [9, 10]. Cold environment is known to alter the composition of the gut microbiota, which in turn affects the production of short-chain fatty acids (SCFAs) [10]. At present, investigations into the correlation between cold environment and the gut microbiota are focused primarily on mammalian animals, whereas studies involving humans are relatively limited [11]. Our previous research demonstrated that cold season increases platelet aggregation and inflammatory cytokine levels in high-risk individuals, exacerbates gut microbiota dysbiosis and intestinal barrier dysfunction, and thereby elevates the risk of ischemic stroke [5, 12].

Based on this background, we hypothesize that gut microbiota dysbiosis associated with cold season may exacerbate the occurrence and progression of ischemic stroke. This study aimed to characterize the gut microbiota associated with cold-season-onset ischemic stroke, investigate its associations with clinical parameters, and explore its potential role in stroke pathogenesis. The findings may provide a theoretical foundation for identifying novel therapeutic targets for AIS.

## Methods

### Population study design

This prospective research was performed from November 2022 to August 2023 in the Department of Neurology, the Second Hospital of Tianjin Medical University. The inclusion criteria were as follows: (a) diagnosis of first-ever AIS and hospitalization within 72 h of stroke onset; (b) age between 50 and 80 years; (c) no significant changes in residence and dietary habits in the past month; and (d) informed consent obtained from the patients or their legal representative. Exclusion criteria: (a) use of antibiotics, prebiotics, or probiotics in the past month; (b) history of gastrointestinal surgery or gastrointestinal disorders; (c) concurrent neurological diseases (such as Parkinson’s disease or Alzheimer’s disease) or mental illnesses; (d) presence of a malignant tumor or poor general condition; (e) dietary restrictions, vegetarianism, or a body mass index (BMI) ≥ 28 kg/m² or ≤ 18.5 kg/m²; (f) failure to provide stool samples; and (g) loss to follow-up.

This study defined the “cold season” based on commonly used criteria in environmental and epidemiological research, specifically spanning from November to March of the following year [13–15]. Accordingly, patients with AIS whose onset occurred between November 1, 2022, and March 31, 2023, were assigned to the cold-season onset ischemic stroke (CIS) group, while the remaining patients were included in the non-cold-season onset ischemic stroke (NCIS) group. Written informed consent was obtained from all participants prior to sample collection.

### Demographics and clinical data collection, follow-up and outcome assessment

The detailed information on demographic and clinical data collection is provided in the supplementary materials. Stroke severity was assessed using the National Institute of Health Stroke Scale (NIHSS). In this study, an NIHSS score ≤ 5 was defined as mild stroke [16–18]. The participants involved in the study were monitored at 3 months poststroke by an experienced neurologist who was blinded to the study design via either in-person or telephone interviews. All participants were successfully followed up. Patients were grouped into two categories according to the modified Rankin scale (mRS) score: the poor prognosis group (mRS 3–6) and the good prognosis group (mRS 0–2).

### Quantification of serum inflammatory indicators

Serum concentrations of tumor necrosis factor alpha (TNF-α), interleukin-8 (IL-8), interleukin-12 (IL-12), and interleukin-1 beta (IL-1β) were measured using commercially available enzyme-linked immunosorbent assay (ELISA) kits (Elabscience, Wuhan, China) according to the manufacturer’s instructions.

### Human fecal collection and 16 S rRNA sequencing [19]

All participants provided the first fresh fecal sample after hospital admission, prior to any antibiotic administration. Samples were collected using sterile, DNA-free fecal collection tubes and processed in a Class II biological safety cabinet within 30 min of defecation. Each sample was divided into two portions. One portion was immediately snap-frozen in liquid nitrogen and stored at − 80 °C for 16 S rRNA gene sequencing. Detailed procedures for 16 S rRNA gene sequencing are described in the supplementary materials. The other portion was homogenized with a pre-cooled cryoprotective solution at a ratio of 1:5 (w/v). The cryoprotective solution consisted of brain heart infusion (BHI) broth supplemented with 20% sterile glycerol and 0.1% freshly prepared L-cysteine (all from Shanghai Sanjiang Pharmaceutical Co., Ltd., China). All components were autoclaved or filter‑sterilized prior to mixing. The homogenate was aliquoted into single-use sterile cryotubes and stored at − 80 °C for subsequent fecal microbiota transplantation (FMT).

### Experimental animals

Male C57BL/6J mice (8–10 weeks old, 18–22 g) were purchased from Charles River Laboratories and were raised with a set temperature (22 °C ± 2 °C) and humidity (40–60%), a 12/12-h light/dark cycle, and free access to food and water for one week before the experiment. Subsequently, the mice were randomly divided into 3 groups (*n* = 7): the NC group, the CIS-FMT group, and the NCIS-FMT group. All mice were treated according to the protocols approved by the guidelines for ethical review of the welfare of experimental animals.

### FMT

To deplete the endogenous intestinal microbiota, all mice received an antibiotic cocktail for 7 days following a one-week acclimation period. The cocktail consisted of ampicillin (1 g/L), neomycin (1 g/L), metronidazole (1 g/L), and vancomycin (500 mg/L) (all from Sangon Biotech, Shanghai, China), administered daily via oral gavage (200 µL/mouse) alongside continuous access to drinking water supplemented with the same antibiotics. The antibiotic-containing water was refreshed every 3–4 days to ensure sustained efficacy [20]. The preparation of fecal suspension and FMT procedure were performed as previously described [21, 22], with detailed methods provided in the supplementary materials. The two groups of FMT mice were enemas (200 µL/each) using the fecal suspension from CIS and NCIS patients daily for one week. The NC group mice were enemas with an equal volume of PBS as a negative control. After the last gavage, mice were maintained under standard laboratory conditions for an additional 7 days to allow microbial stabilization.

### Middle cerebral artery occlusion (MCAO) and behavioral testing

The MCAO procedure was performed as previously described [7], with detailed methods provided in the supplementary materials. The modified neurological severity score (mNSS) was assessed 24 h after MCAO by two independent investigators blinded to the study design. The mean value was used for statistical analysis. The score ranges from 0 to 18, with 0 indicating normal neurological function and 18 representing the most severe neurological deficits [23]. Mice were excluded if (a) surgical duration exceeded 15 min, (b) death occurred within 24 h, (c) mNSS was < 1 at 24 h post-surgery, or (d) severe intraoperative bleeding or inability to successfully insert the suture plug. Of the 21 mice initially enrolled (*n* = 7 per group), one mouse in the CIS-FMT group and one in the NCIS-FMT group died within 24 h after MCAO surgery and were excluded from the final analysis. Additionally, two mice in the NC group and one mouse each in the CIS-FMT and NCIS-FMT groups were excluded because their mNSS score was less than 1 at 24 h post-surgery.

### Fecal metagenomics and metabolomics in FMT-treated mice

Twenty-four hours after MCAO, fresh fecal samples were collected from mice in the CIS-FMT and NCIS-FMT groups for subsequent metagenomic and untargeted metabolomics analyses. Detailed methodologies are described in the supplementary materials.

### Serum biochemical analysis, cerebral infarction measurement, and tissue immunohistochemistry

Mice were deeply anesthetized, and blood samples were collected via the orbital venous plexus. Serum was obtained after centrifugation for biochemical analysis. Serum levels of lipopolysaccharide (LPS) and lipopolysaccharide-binding protein (LBP) were measured using ELISA kits (Elisalab) according to the manufacturer’s instructions.

Euthanasia was then performed via intraperitoneal injection of sodium pentobarbital (150 mg/kg). This method ensured complete unconsciousness and minimized pain and distress, in accordance with institutional and national animal welfare guidelines. Following euthanasia, brain and colon tissues were harvested for further analysis. The detailed procedure for infarct volume measurement is provided in the supplementary materials [24].

After fixation in 4% paraformaldehyde and paraffin embedding as previously described, mouse colon tissue samples were sectioned into 4-µm-thick slices [25]. The detailed procedure of IHC staining and semi-quantitative analysis is provided in the supplementary materials [12].

### Bioinformatics analysis

Alpha diversity was compared between groups using the Wilcoxon rank-sum test, while beta diversity was evaluated by principal coordinate analysis (PCoA) based on Bray–Curtis dissimilarity. Relative abundances at the phylum, family, and genus levels were calculated to characterize microbial community differences. Differential taxa were identified using the linear discriminant analysis effect size (LEfSe) method, which applies nonparametric Kruskal–Wallis tests and LDA to detect significantly enriched taxa. In addition, the Wilcoxon rank-sum test was used to assess differential abundances of dominant phyla, families, and genera.

Spearman correlation analysis was performed to examine associations between clinical parameters and the top 15 genera, and results were visualized in a heatmap. Receiver operating characteristic (ROC) curves were generated to assess the predictive performance of representative microbial genera for patient prognosis. Candidate genera used in the prognostic prediction model were selected through a three-step screening process: (1) genera ranked within the top 15 in relative abundance; (2) taxa significantly enriched in LEfSe analysis (LDA > 3.5, *p* < 0.05); and (3) genera significantly correlated with baseline NIHSS and/or 90-day mRS scores.

Functional profiling was conducted using the Phylogenetic Investigation of Communities by Reconstruction of Unobserved States 2 (PICRUSt2) pipeline [26], with pathway annotation referenced to the Kyoto Encyclopedia of Genes and Genomes (KEGG) database [27]. All bioinformatic analyses were performed using the online tools provided by the Majorbio Cloud Platform.

### Statistical analysis

The normality of data distribution was assessed using the Kolmogorov–Smirnov test. Continuous variables were expressed as the mean ± standard deviation (SD) or median with interquartile range (IQR), depending on distribution characteristics. Comparisons between two groups were performed using independent-samples t-tests for normally distributed variables or nonparametric tests when appropriate. Comparisons among multiple groups were conducted using one-way analysis of variance (ANOVA) followed by Tukey’s post hoc test. Categorical variables were presented as counts and percentages and compared using the χ² test. Statistical analyses were conducted using GraphPad Prism 9.5.1 and SPSS 25.0. A two-sided *p*-value < 0.05 was considered statistically significant.

## Results

### Baseline characteristics of the participants

**Fig. 1:**
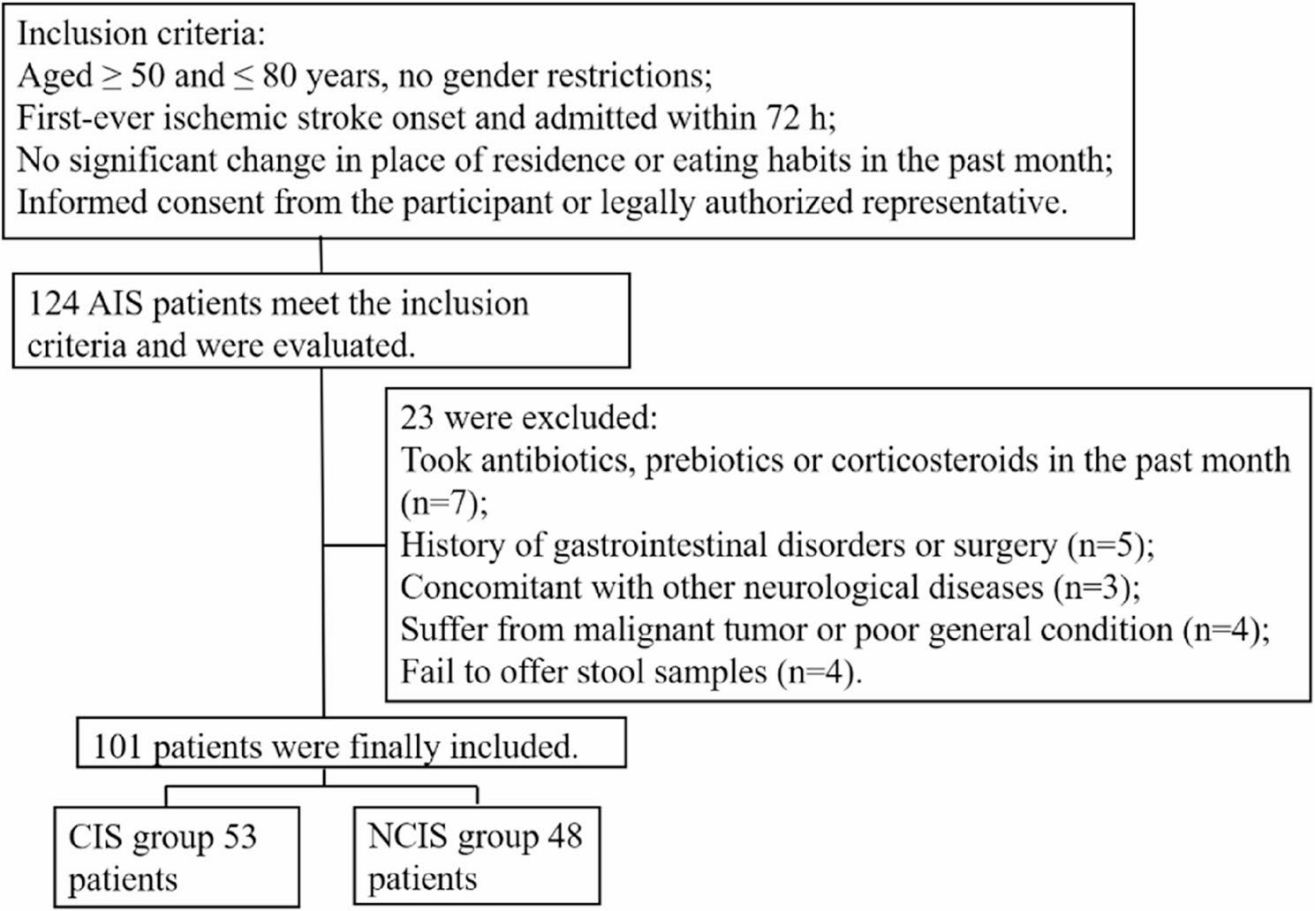
Flowchart of the admission process

During the study period, 124 patients met the inclusion criteria. After applying the exclusion criteria, a total of 101 patients were included in the final analysis (Fig. 1). Compared to the NCIS group (*n* = 48), the CIS group (*n* = 53) showed significantly higher WBC counts, NLRs, SIRIs, LDL-C, TNF-α, baseline NIHSS scores, and 90-day mRS scores (*p* < 0.05). Detailed characteristics are presented in Table 1.

**Table 1:** Characteristics of the CIS and NCIS groups

Parameter	Total (*n* = 101)	CIS group (*n* = 53)	NCIS group (*n* = 48)	*p*
Demographics				
Age, y	68 (59, 74)	68 (59, 75)	67 (57, 74)	0.404
Male	61 (60.4%)	30 (56.6%)	31 (64.6%)	0.413
BMI, kg/m^2^	22.72 ± 2.74	23.10 ± 2.53	22.30 ± 2.92	0.530
Smoking	51 (50.5%)	28 (52.8%)	23 (47.9%)	0.622
Drinking	30 (29.7%)	12 (22.6%)	18 (37.5%)	0.103
Vascular risk factors				
Hypertension	65 (64.4%)	35 (66.0%)	30 (62.5%)	0.711
Diabetes mellitus	33 (32.7%)	18 (34.0%)	15 (31.3%)	0.772
Hyperlipidemia	54 (53.5%)	28 (52.8%)	26 (54.2%)	0.893
Coronary heart disease	26 (25.7 %)	13 (24.5%)	13 (27.1%)	0.769
Atrial fibrillation	10 (9.9%)	5 (9.4%)	5 (10.4%)	0.565
Dietary habits				0.316
Vegetarian-based diet‌	39 (38.6%)	24 (45.3%)	15 (31.3%)	
Meat-based diet	34 (33.7%)	15 (28.3%)	19 (39.6%)	
Mixed-based diet‌	28 (27.7%)	14 (26.4%)	14 (29.2%)	
SBP, mmHg	150.5 ± 20.0	151.85 ± 20.87	149.06 ± 19.03	0.486
DBP, mmHg	88.5 ± 13.4	88.19 ± 13.13	88.90 ± 13.78	0.792
Baseline NIHSS scores	3 (1, 6)	5 (2, 8)	2.5 (1, 3)	0.001^*^
Stroke severity				0.001^*^
mild stroke	64 (63.4%)	25 (47.2%)	39 (81.3%)	
non-mild stroke	37 (36.6%)	28 (52.8%)	9 (18.7%)	
Laboratory tests				
WBC, ×10^9^/L	7.10 (5.99, 8.48)	7.38 (6.49, 9.09)	7.08 (5.62, 7.95)	0.040^*^
NLR	2.48 (1.88, 3.38)	2.90 (2.07, 4.12)	2.32 (1.75, 2.87)	0.008^*^
SII	515 (393.5, 816)	545.0 (409.5, 926.0)	474.5 (314.5, 724.0)	0.161
SIRI	1.04 (0.73, 1.57)	1.19 (0.80, 1.87)	0.91 (0.70, 1.31)	0.032^*^
TC, mmol/L	4.76 ± 1.23	4.83 ± 1.26	4.68 ± 1.20	0.534
TG, mmol/L	1.53 ± 0.77	1.55 ± 0.89	1.51 ± 0.62	0.776
HDL-C, mmol/L	1.12 ± 0.31	1.13 ± 0.36	1.12 ± 0.25	0.819
LDL-C, mmol/L	3.06 ± 1.00	3.34 ± 0.97	2.74 ± 0.93	0.002^*^
FBG, mmol/L	6.45 ± 2.90	6.79 ± 3.50	6.06 ± 2.01	0.196
HbA1c	6.1 (5.7, 7.2)	6.10 (5.65, 7.15)	6.15 (5.70, 7.38)	0.820
HCY, μmol/L	13.32(10.75, 16.97)	13.11 (11.03, 20.11)	13.39 (10.22, 16.48)	0.301
IL-1β, pg/ml	14.39 ± 2.92	14.90 ± 2.83	13.82 ± 2.94	0.063
IL-8, pg/ml	398.95 (222.71, 654.66)	433.5 (264.1, 712.6)	384.5 (163.1, 573.9)	0.075
IL-12, pg/ml	6.99 (5.40, 9.54)	7.4 (6.3, 10.3)	6.3 (5.0, 9.2)	0.052
TNF-α, pg/ml	12.85 ± 5.30	13.90 ± 4.55	11.68 ± 5.85	0.038^*^
mRS scores	1 (1, 3)	2 (1, 4)	1 (1, 2)	0.028^*^
Poor prognosis	29 (28.7%)	20 (37.7%)	9 (18.8%)	0.035^*^

*BMI* Body mass index, *SBP* Systolic blood pressure, *DBP* Diastolic blood pressure, *NIHSS* National Institutes of Health Stroke Scale, *WBC *White blood cell, *NLR* Neutrophil-to-lymphocyte ratio, *SII* Systemic Immune-Inflammation Index, *SIRI* Systemic Inflammatory Response Index, *TC* Total cholesterol, *TG* Triglyceride, *HDL-C* High-density lipoprotein cholesterol, *LDL-C* Low-density lipoprotein cholesterol, *FBG* Fasting blood glucose, *HbA1c* Glycated hemoglobin, *HCY* Homocysteine, *IL* Interleukin, *TNF* Tumor necrosis factor, *mRS* Modified Rankin scale. Data in the table are expressed as mean ± standard deviation, median (*P*_25_, *P*_75_), and cases (%)

### Gut microbiota diversity and composition in CIS and NCIS patients

**Fig. 2:**
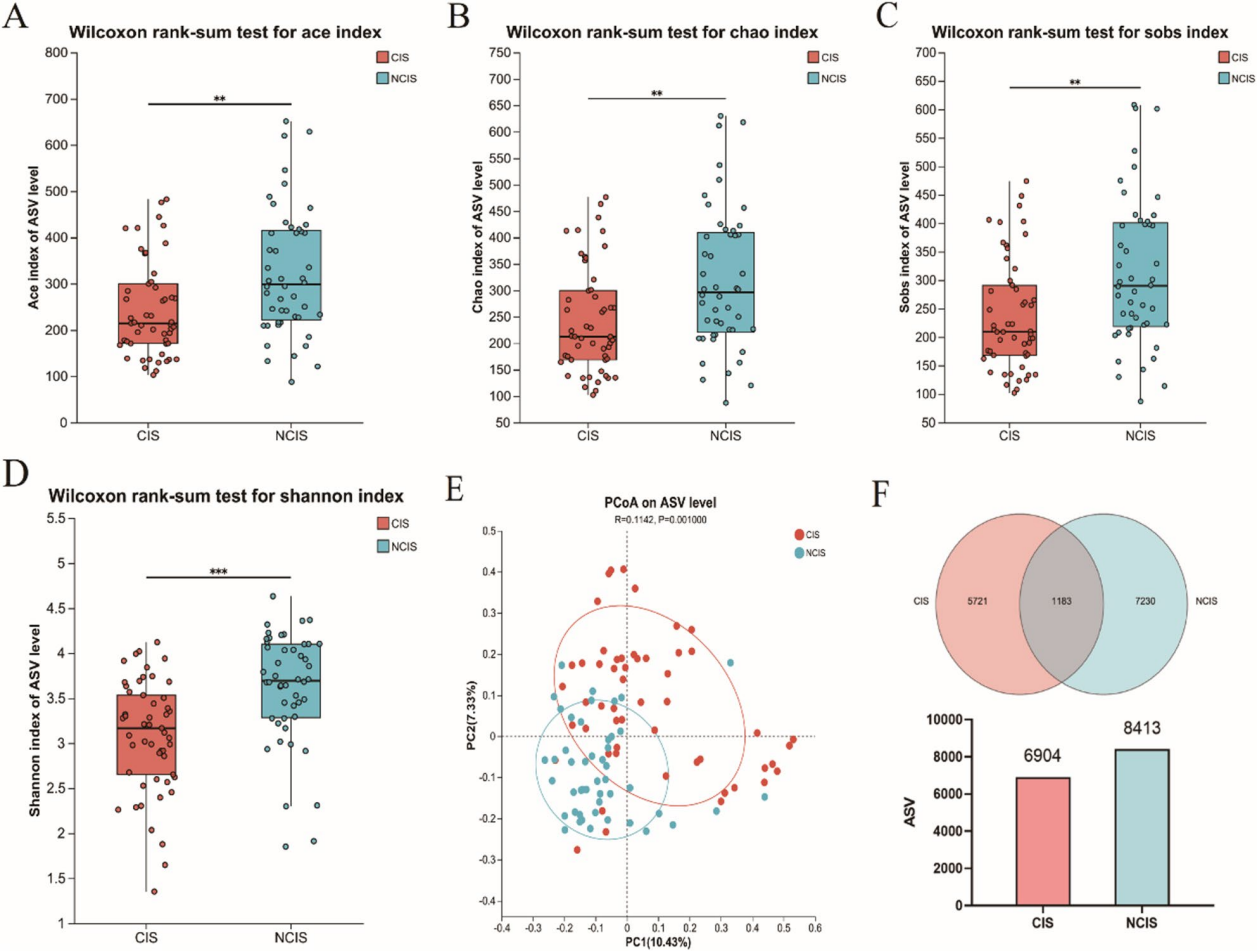
Comparison of gut microbiota diversity between CIS and NCIS patients. **A–D** ACE, Chao1, Observed features and Shannon index; (**E**) PCoA based on Bray Curtis distances demonstrated a clear separation in microbial community structure between the two groups; (**F**) The Venn diagram shows the number of unique ASVs and shared (gray) ASVs for the CIS group (light red) and the NCIS group (light blue). CIS, cold-season onset ischemic stroke; NCIS, non-cold-season onset ischemic stroke. PCoA, Principal Coordinate Analysis. ^**^p < 0.01, ^***^p < 0.001

Gut microbiota diversity differed significantly between the CIS and NCIS groups (Fig. 2). The CIS group exhibited significantly lower α-diversity than the NCIS group, as measured by the ACE, Chao1, Observed features, and Shannon indices (*p* < 0.05, Fig. 2A-D). Principal Coordinate Analysis (PCoA) based on Bray Curtis distances demonstrated a clear separation in microbial community structure between the two groups (ANOSIM: *R* = 0.114, *p* = 0.001, Fig. 2E). Consistently, a Venn diagram showed that while 1,183 ASVs were shared, the CIS group possessed fewer unique amplicon sequence variants (ASVs) (5,721) than the NCIS group (7,230), further supporting a loss of microbial richness in the CIS group (Fig. 2F).

The composition of the gut microbiota in the two groups at the phylum, family, and genus levels, along with the corresponding comparative analyses, is shown in Figure S1.

### Differential microbiota between CIS and NCIS patients

**Fig. 3:**
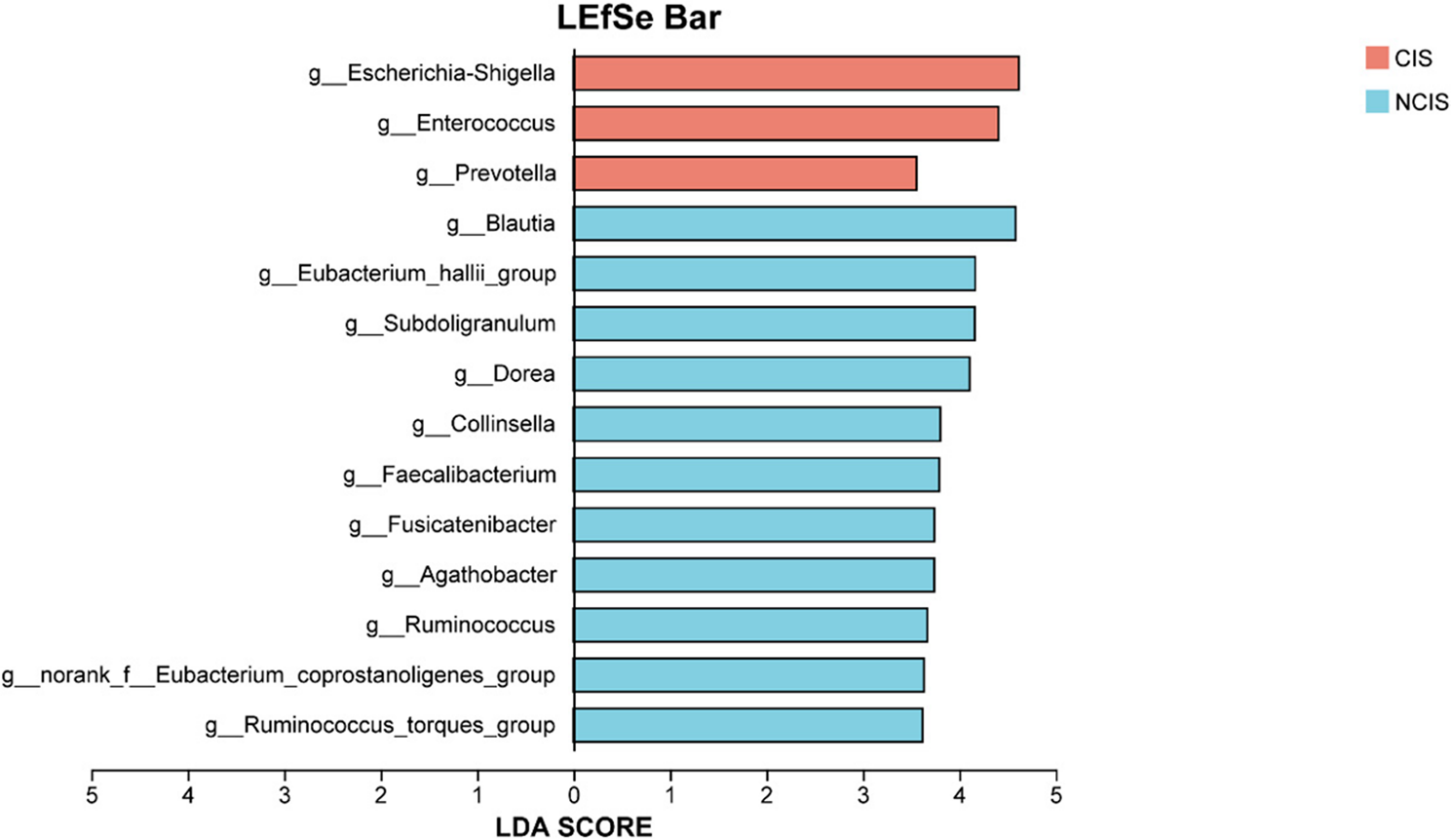
Analysis of characteristic microbiota between CIS and NCIS patients. A distribution diagram of LDA scores highlights the gut microbiota at the genus level with significant differentiation (LDA > 3.5)

To further identify the most differentially abundant taxa between the CIS and NCIS groups, LEfSe analysis was performed. As depicted in Fig. 3, 14 taxa with LDA > 3.5 and *p* < 0.05 were identified as distinctive microbiota. In the CIS group, the genera exhibiting significant differences predominantly included *Escherichia-Shigella*,* Enterococcus*, and *Prevotella*. Conversely, the NCIS group exhibited a notable presence of *Blautia*,* Eubacterium_hallii_group*,* Subdoligranulum*,* Dorea*,* Collinsella*,* Faecalibacterium*,* Fusicatenibacter*,* Agathobacter*,* Ruminococcus*,* norank_f_Eubacterium_coprostanoligenes_group*, and *Ruminococcus_torques_group*.

### PICRUSt2 functional prediction of CIS and NCIS patients

**Fig. 4:**
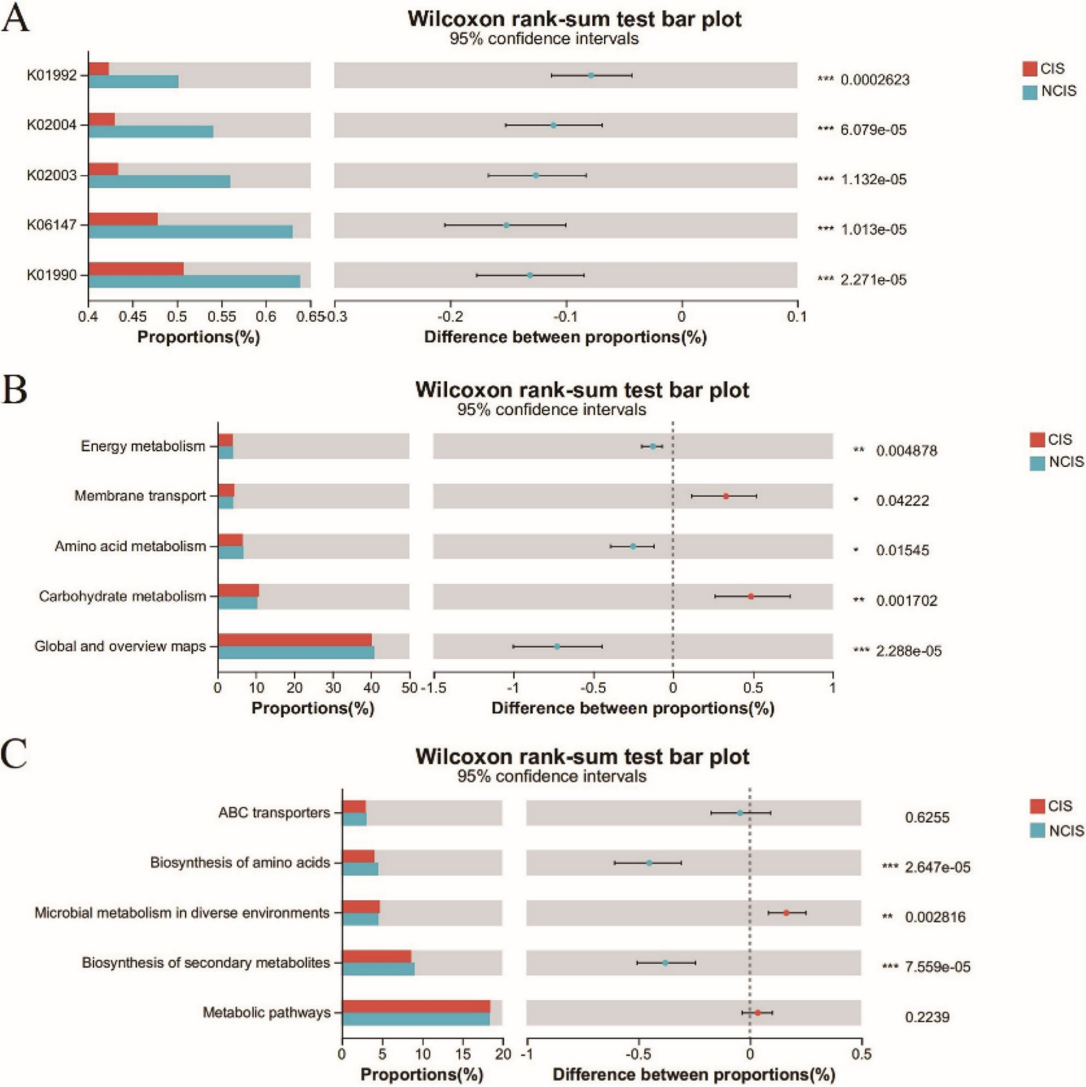
Functional prediction of microbial communities between CIS and NCIS patients. **A** K01992: Carbohydrate metabolism; K02004: Membrane transport; K02003: Membrane transport/Energy-dependent transport; K06147: Energy metabolism/Carbon metabolism, K01990: Amino acid metabolism; (**B**) KEGG level 2; (**C)** KEGG level 3. ^*^p < 0.05, ^**^p < 0.01, ^***^p < 0.001

PICRUSt2 functional prediction combined with KEGG database metabolic pathway analysis predicted differentially abundant metabolic pathways between the CIS and NCIS groups. When integrated with the KEGG Orthology (KO) database, PICRUSt2 predicted that the microbial function in the CIS group was mainly related to the decline of carbohydrate metabolism, membrane transport, energy metabolism, and amino acid metabolism (*p* < 0.001, Fig. 4A). When combined with the KEGG level 2 database, pathway analysis revealed a reduction in energy metabolism (*p* < 0.01) and amino acid metabolism (*p* < 0.05) in the CIS group (Fig. 4B). By integrating deeper KEGG level 3 data, it was further confirmed that the microbial community in the CIS group presented a reduction in amino acid biosynthesis (*p* < 0.001, Fig. 4C).

### Associations between microbiota and clinical parameters in the human study

**Fig. 5:**
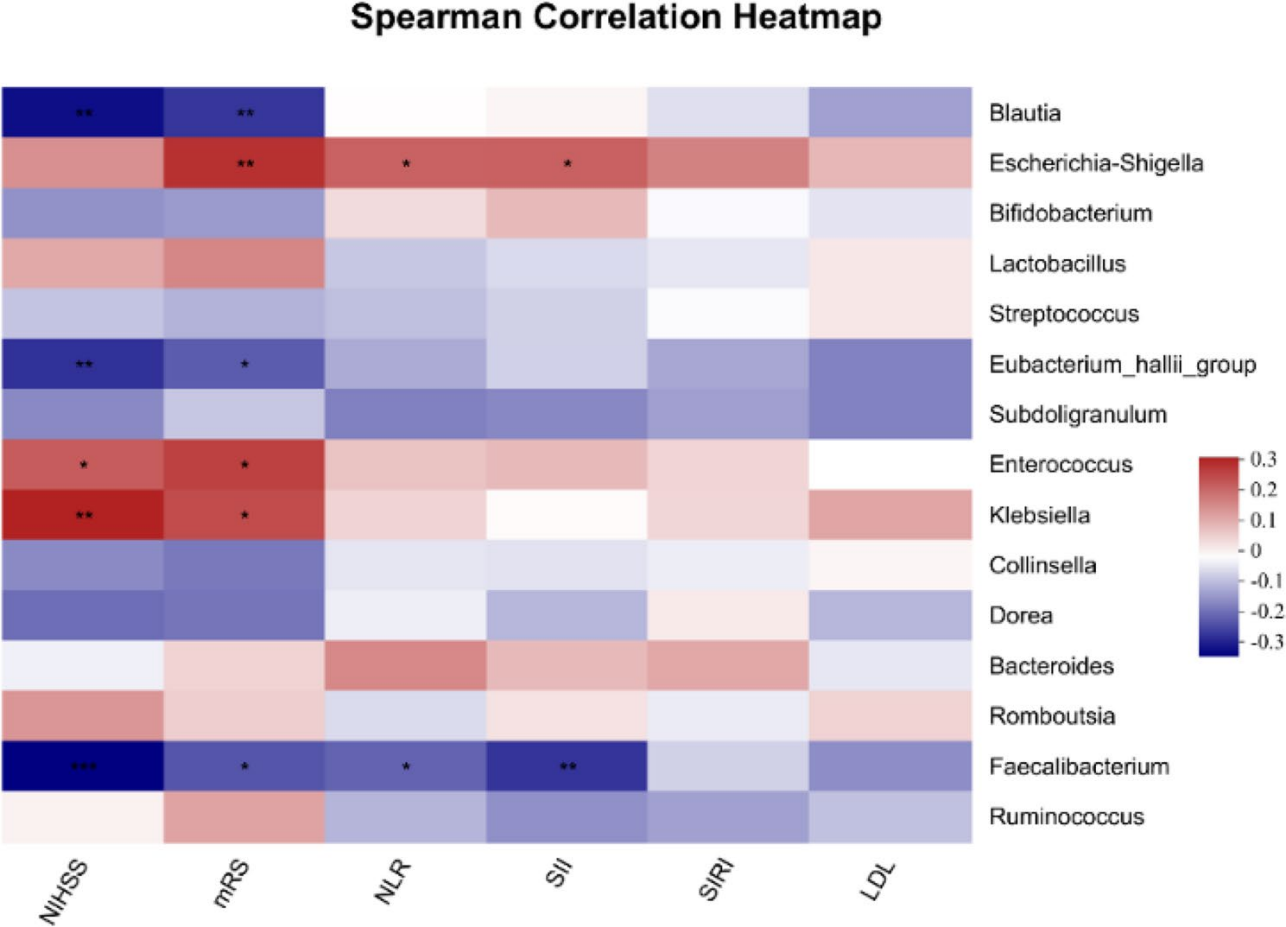
Heatmap of Spearman correlation analysis between the top 15 genera and clinical parameters. Red grids indicate positive correlations, and blue grids indicate negative correlations. A deeper red or blue color indicates higher correlation values. ^*^p < 0.05, ^**^p < 0.01, ^***^p < 0.001

The correlation between the top 15 genera and clinical indicators was explored using the Spearman correlation analysis (Fig. 5). Baseline NIHSS scores were positively associated with *Enterococcus* (*r* = 0.214, *p* = 0.031) and *Klebsiella* (*r* = 0.296, *p* = 0.003) but inversely related to *Blautia* (*r* = -0.311, *p* = 0.002), *Eubacterium_hallii_group* (*r* = -0.268, *p* = 0.007), and *Faecalibacterium* (*r* = -0.328, *p* < 0.001). The 90-day mRS scores were positively correlated with *Escherichia-Shigella* (*r* = 0.275, *p* = 0.005), *Enterococcus* (*r* = 0.253, *p* = 0.011) and *Klebsiella* (*r* = 0.237, *p* = 0.017), but inversely correlated with *Blautia* (*r* = -0.262, *p* = 0.008), *Eubacterium_hallii_group* (*r* = -0.215, *p* = 0.031), and *Faecalibacterium* (*r* = -0.224, *p* = 0.025). Furthermore, *Escherichia-Shigella* was positively correlated with the NLR (*r* = 0.206, *p* = 0.038) and SII (*r* = 0.207, *p* = 0.038), whereas *Faecalibacterium* was inversely related to the NLR (*r* = -0.207, *p* = 0.038) and SII (*r* = -0.265, *p* = 0.008).

### Associations between microbiota and inflammatory indicators in the human study

Figure S2 shows that among the top 15 genera, IL-1β was positively associated with *Escherichia-Shigella* (*r* = 0.203, *p* = 0.042), whereas IL-12 was positively correlated with *Enterococcus* (*r* = 0.270, *p* = 0.006). Conversely, IL-8 was inversely correlated with *Dorea* (*r* = -0.260, *p* = 0.009), and TNF-α exhibited the opposite relationship with *Faecalibacterium* (*r* = -0.242, *p* = 0.015).

### Predictive performance of gut microbiota in the human study

The participants were classified into two groups on the basis of the 90-day mRS scores: the poor prognosis group (*n* = 29) and the good prognosis group (*n* = 72). Based on LEfSe and correlation analyses, five genera among the top 15 most abundant were identified as potential biomarkers for predicting functional outcomes in patients: *Escherichia-Shigella*, *Blautia*, *Enterococcus*, *Eubacterium_hallii_group*, and *Faecalibacterium*. As presented in Fig. S3A, the combination of 5 genera exhibited strong predictive ability for adverse outcomes (*p* < 0.001, AUC = 0.848, 95% CI: 0.756–0.940). Similarly, the combination of *Escherichia-Shigella* and *Enterococcus* also demonstrated significant predictive power for poor prognosis (*p* < 0.001, AUC = 0.810, 95% CI: 0.706–0.914; Fig. S3B). Moreover, *Escherichia-Shigella* (*p* = 0.001, AUC = 0.707, 95% CI: 0.585–0.829) and *Enterococcus* (*p* = 0.001, AUC = 0.707, 95% CI: 0.588–0.826) were independently distinguished between the two groups (Fig. S3C). However, *Blautia* (*p* = 0.005, AUC = 0.681, 95% CI: 0.566–0.796), *Eubacterium_hallii_group* (*p* = 0.072, AUC = 0.615, 95% CI: 0.492–0.738) and *Faecalibacterium* (*p* = 0.031, AUC = 0.638, 95% CI: 0.516–0.759) were not strong predictors of poor prognosis (Fig. S3D).

### Cold-related gut dysbiosis may influence brain injury, intestinal barrier dysfunction and inflammatory factor levels in MCAO mice

**Fig. 6:**
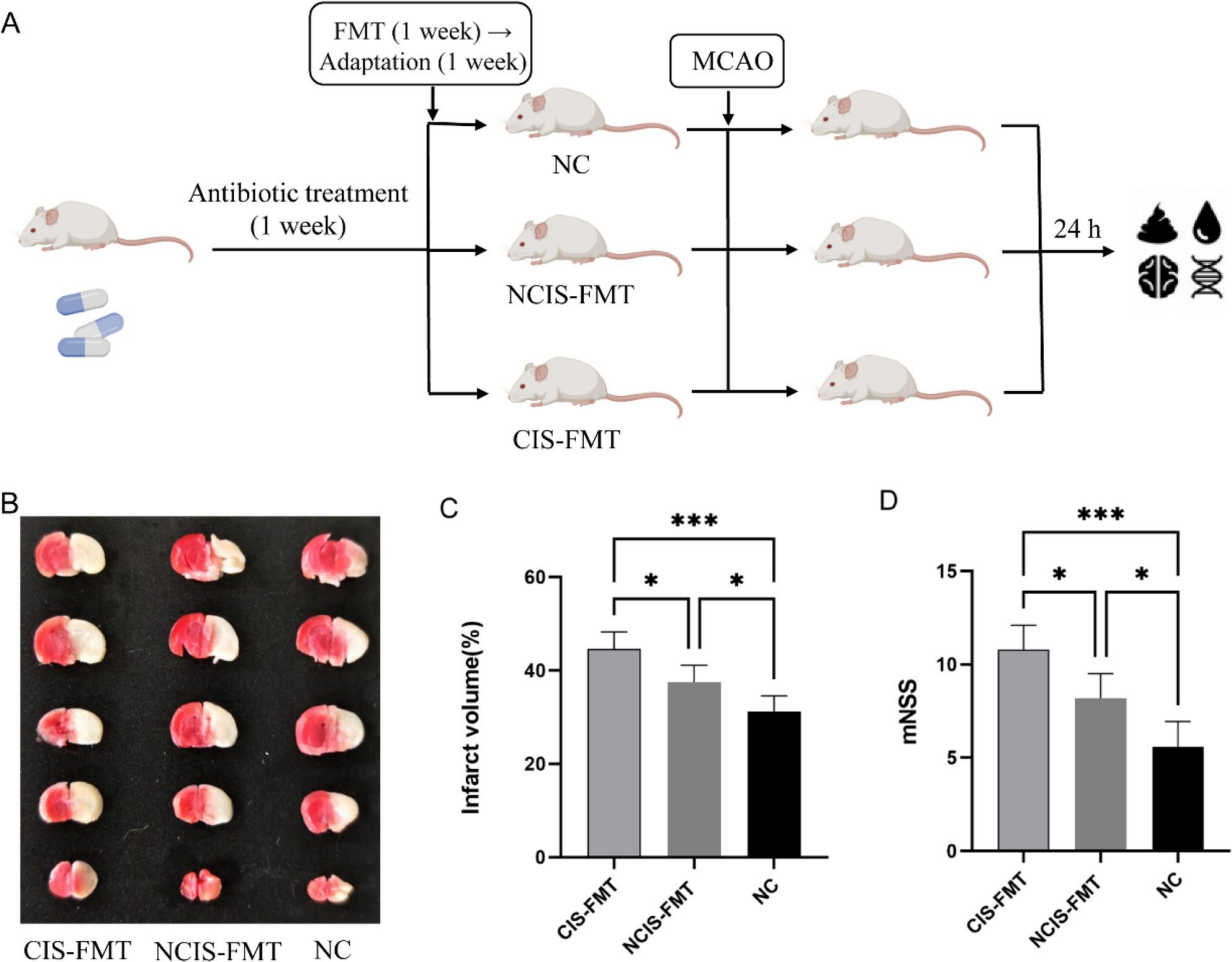
FMT may influence brain injury in MCAO mice. **A** Animal experimental protocol. **B** Representative TTC-stained brain sections. **C–D** Quantification of cerebral infarct volume and mNSS scores of the 3 groups (*n* = 5 per group). CIS, cold-season onset ischemic stroke; NCIS, non-cold-season onset ischemic stroke; NC, normal controls; FMT, fecal microbiota transplantation. ^*^p < 0.05, ^***^p < 0.001

**Fig. 7:**
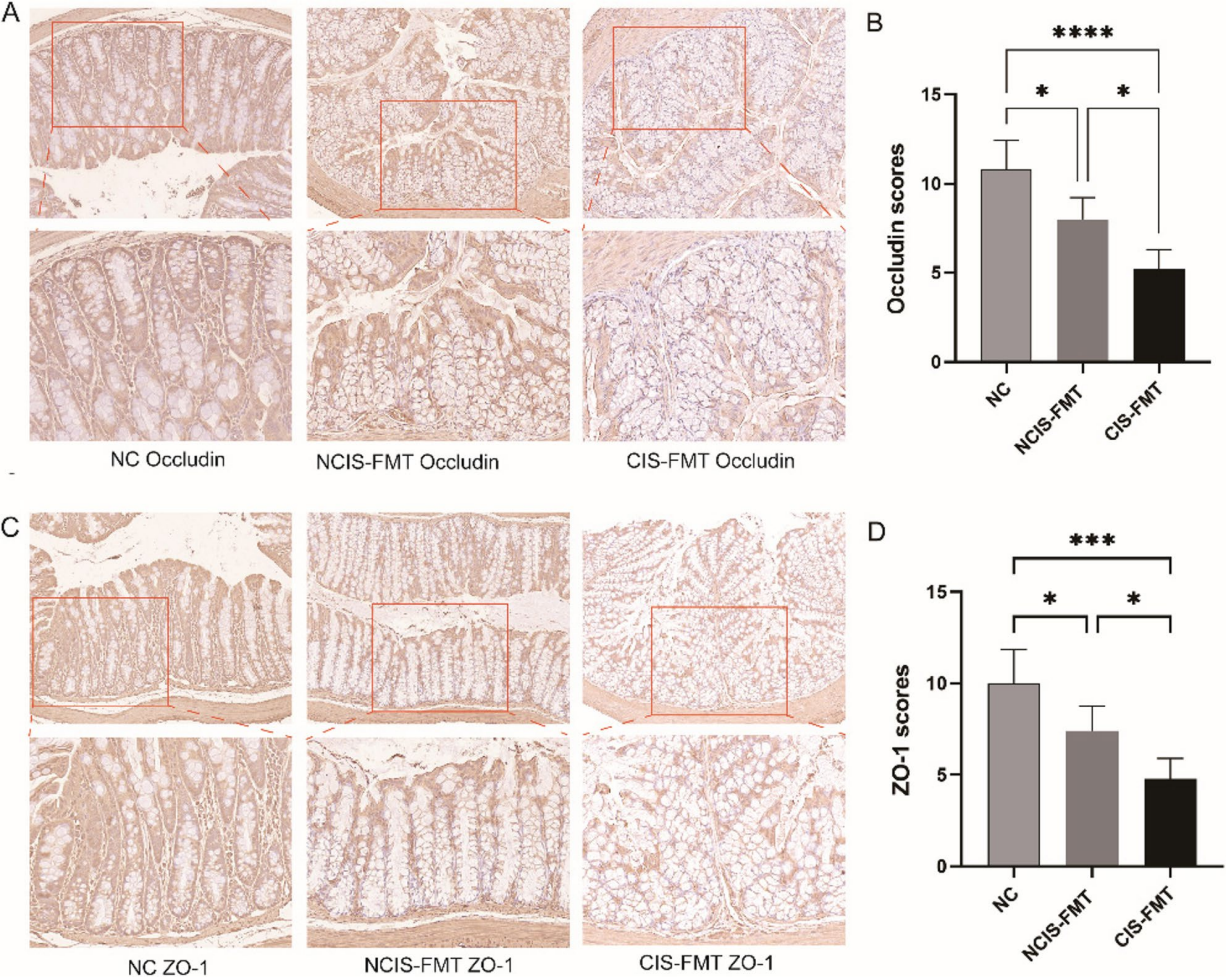
Immunohistochemical staining of Occludin and ZO-1 in the 3 groups of mice. Representative IHC images (**A**,** C**) and semi-quantitative analysis (**B**,** D**) of Occludin and ZO-1 (magnifications: 200× for upper panels and 400× for lower panels). Data are presented as mean ± SD (*n* = 5 per group). ^*^p < 0.05, ^***^p < 0.001, ^****^p < 0.0001

**Fig. 8:**
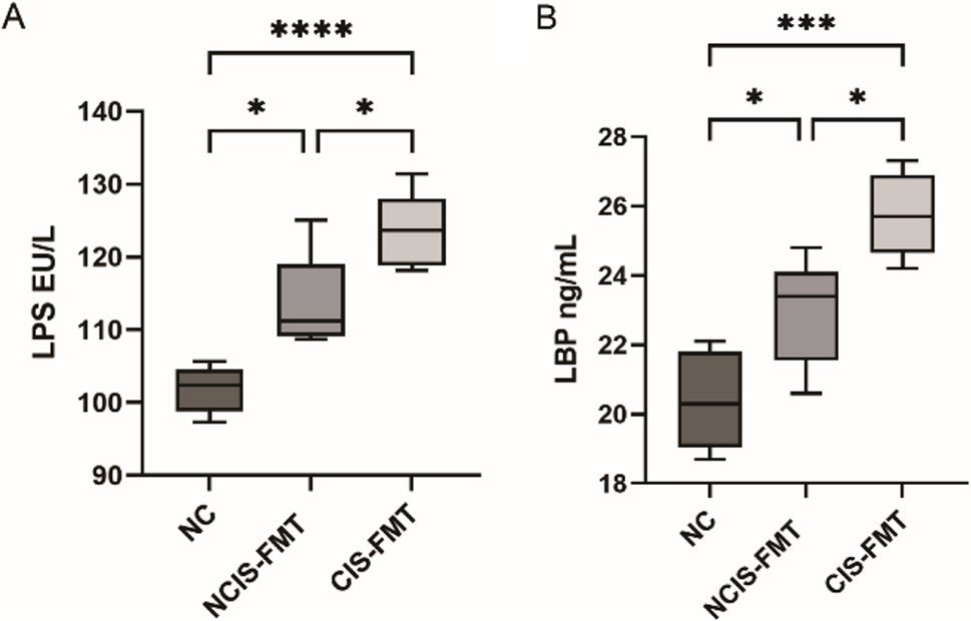
Serum levels of LPS and LBP in the 3 groups of mice. LPS (**A**) and LBP (**B**) levels were significantly elevated in the CIS-FMT group compared with the NCIS-FMT and NC groups. Data are presented as mean ± SD (*n* = 5 per group). ^*^p < 0.05, ^***^p < 0.001, ^****^p < 0.0001

To investigate whether cold-related gut dysbiosis exacerbates post-stroke brain injury and neurological dysfunction, we conducted a FMT experiment (Fig. 6A). Compared with the NC group, the CIS-FMT and NCIS-FMT groups exhibited significantly greater brain infarction and higher mNSS scores, with further increases observed in the CIS-FMT group compared to the NCIS-FMT group (*p* < 0.05, Fig. 6B-D). IHC results showed that the expression levels of Occludin and Zonula Occludens-1 (ZO-1) were significantly decreased in both the CIS-FMT and NCIS-FMT groups compared to the NC group, with a further reduction observed in the CIS-FMT group compared to the NCIS-FMT group (*p* < 0.05, Fig. 7). Moreover, compared to the NC group, pro-inflammatory factors LPS and LBP were significantly elevated in both the CIS-FMT and NCIS-FMT groups, with further increases observed in the CIS-FMT group compared to the NCIS-FMT group (*p* < 0.05, Fig. 8). Taken together, compared with the NCIS group, the gut microbiota of CIS patients may exacerbate brain injury, intestinal barrier dysfunction, and blood inflammatory factor levels in MCAO model mice.

### FMT alters the gut microbiota composition in mice

Analysis of post-FMT metagenomic profiles indicated partial but biologically meaningful engraftment of human-derived microbiota in recipient mice. Key NCIS-enriched genera showed successful colonization, whereas certain CIS-associated taxa exhibited limited expansion, consistent with known host-specific ecological constraints. Detailed colonization patterns are provided in Fig. S4.

**Fig. 9:**
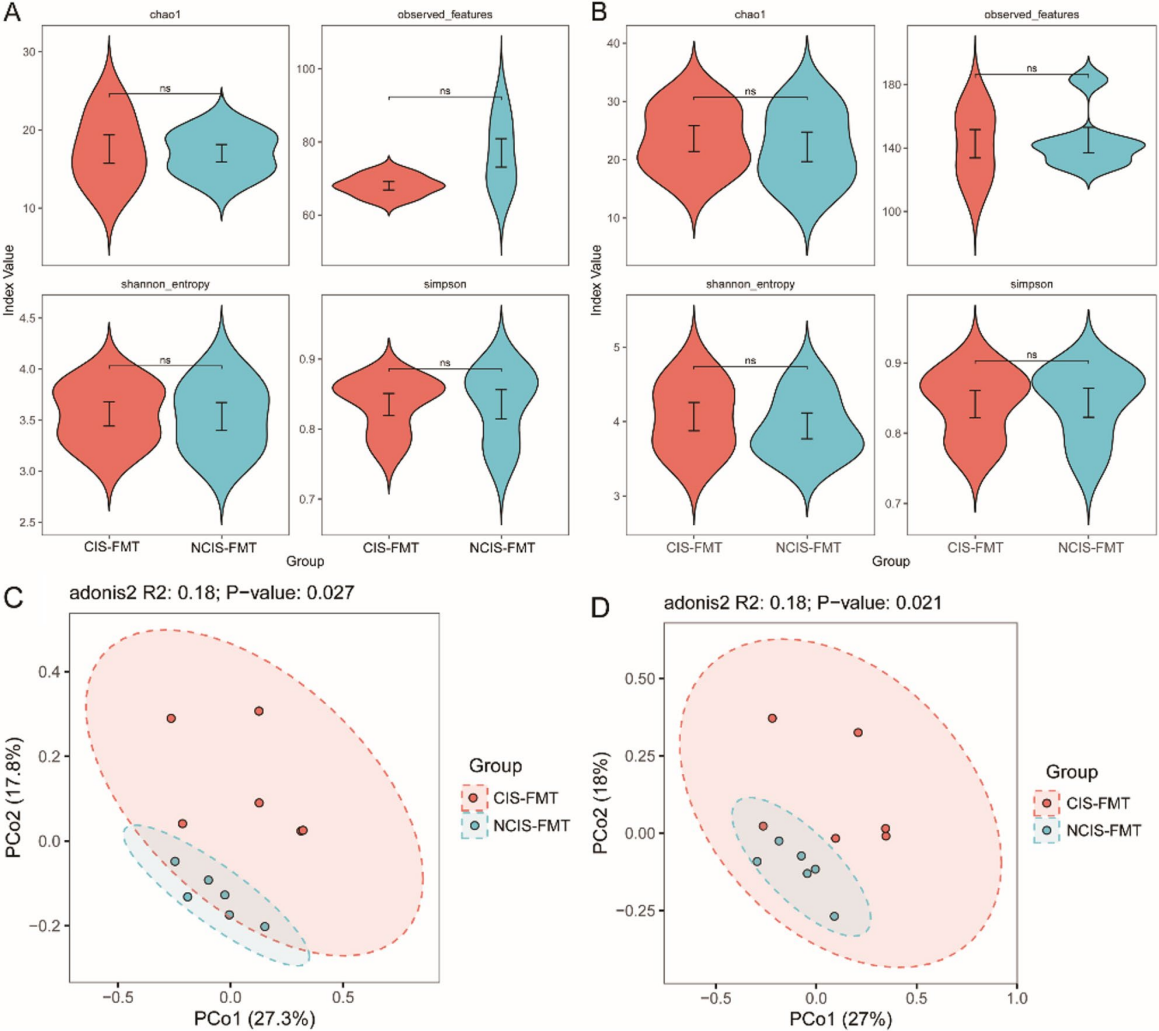
Comparison of gut microbiota diversity between the two groups of mice after FMT. **A-B **α-diversity at genus and species level. **C-D** PCoA based on weighted UniFrac distances at genus and species level. ns, no statistically significant difference

After FMT, there were no significant differences in α-diversity between CIS-FMT and NCIS-FMT mice at either the genus or species level (Fig. 9A-B, *p* > 0.05). PCoA based on weighted UniFrac distances revealed distinct separation of the gut microbiota community structure between the two groups at both the genus (R^2^ = 0.18, *p* = 0.027) and species (R^2^ = 0.18, *p* = 0.021) levels, suggesting a significant difference in the gut microbiota composition of the two groups (Fig. 9C-D).

**Fig. 10:**
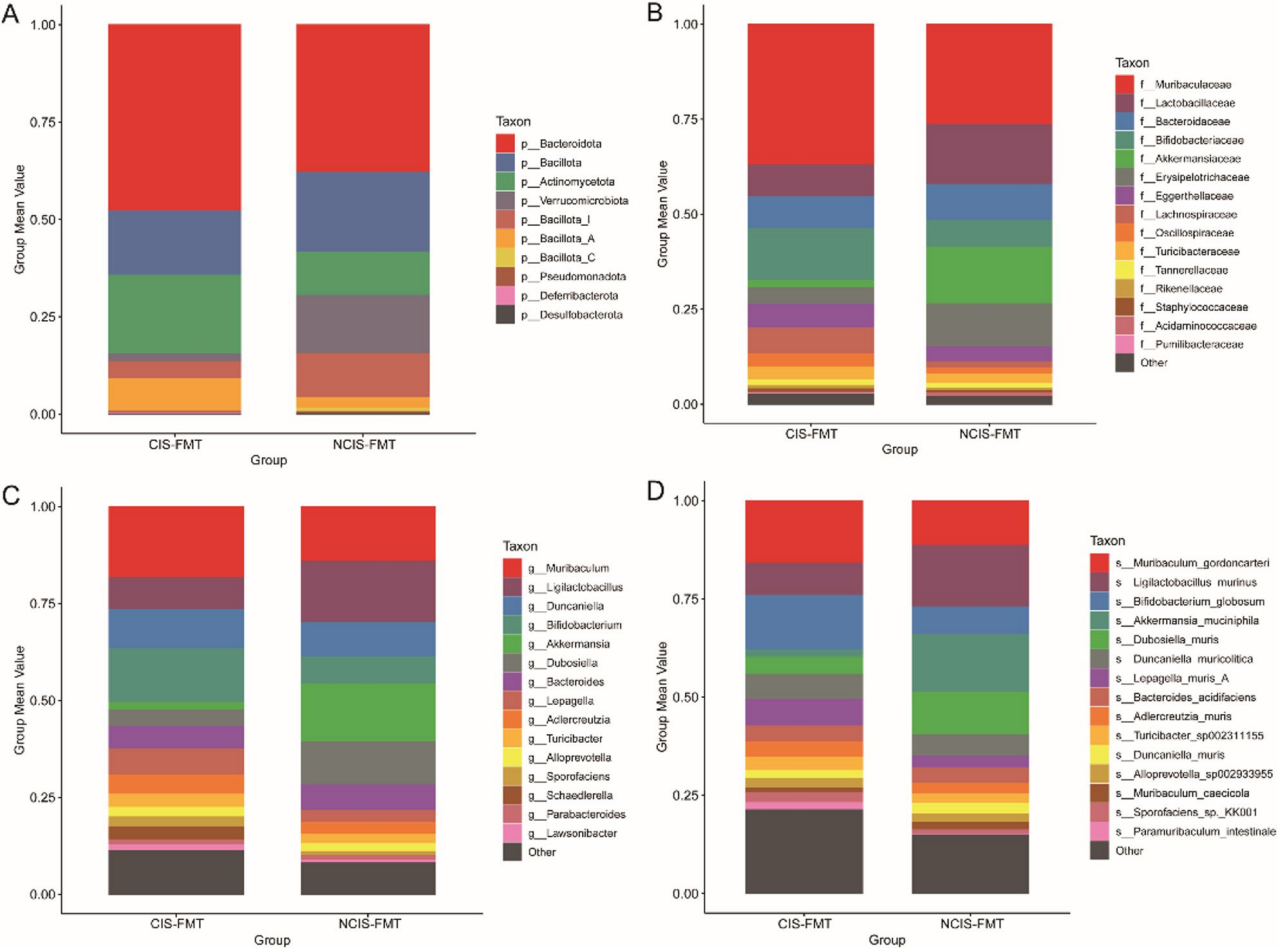
Bar charts showing the relative distribution of gut microbiota at various taxonomic levels in the two groups of FMT-treated mice. **A **phylum level; (**B**) family level; (**C**) genus level; (**D**) species level

Figure 10 show the cumulative bar charts of the relative distributions of the gut microbiota at various taxonomic levels in the two groups of mice after FMT. The rank-sum test of inter-population differences in the major microbial communities is shown in Figure S5.

Next, LEfSe analysis was performed to further assess whether FMT treatment affected the dominant gut microbiota in mice. At the genus level, *Schaedlerella*, *Evtepia*, and *Streptococcus* were enriched in the CIS-FMT group, whereas *Akkermansia*, *Ruminococcus_G*, *Clostridium_AQ*, *Phocaeicola*, *Eubacterium*, *Romboutsia*, and *Collinsella* were enriched in the NCIS-FMT group (LDA > 3, *p* < 0.05; Fig. S6A). At the species level, the CIS-FMT group was enriched with species including *Schaedlerella arabinophila*, *Parabacteroides massiliensis*, *Streptococcus danieliae*, and *Evtetpia gabavorous*. Some of these species have been reported to be associated with inflammatory states or opportunistic pathogenicities. In contrast, the NCIS-FMT group was enriched with symbiotic bacteria such as *Akkermansia muciniphila* and *Eubacterium callanderi*, which are associated with mucosal barrier maintenance or short-chain fatty acid metabolism, as well as other species that may have protective ecological functions (LDA > 2.5, *p* < 0.05; Fig. S6B).

### Correlation between gut microbiota and stroke-related parameters in FMT-treated mice

To explore associations between microbial taxa and stroke-related parameters in FMT-treated mice, correlation analyses were performed. At the genus level, *Akkermansia*, *Clostridium_AQ*, *Eubacterium*, and *Ruminococcus_G* were positively correlated with the intestinal barrier protein ZO-1, whereas *Streptococcus* showed positive correlations with circulating LPS levels and negative correlations with Occludin expression. In contrast, *Romboutsia* was negatively correlated with both LPS levels and mNSS scores (Fig. S7). At the species level, taxa such as *Butyricimonas_virosa*,* Akkermansia muciniphila*,* Clostridium_AQ_innocuum*,* Ruminococcus_G_gauvreauii*,* Parabacteroides gordonii* and *Eubacterium_callanderi* were positively correlated with intestinal barrier protein expression and negatively correlated with systemic inflammatory markers, mNSS scores, and infarct volume. Conversely, species including *Alistipes_cottocaccae*,* Streptococcus danieliae A*, *Schaedlerella_arabinosiphila* and *Evtepia_gabavorous*, which have been previously linked to inflammatory conditions in certain contexts, showed negative correlations with barrier protein levels and positive correlations with inflammatory and stroke severity markers (Fig. S8).

These findings indicate that, in CIS-FMT mice, bacterial taxa enriched in the gut microbiota are associated with disruption of intestinal barrier function and increased inflammatory responses, whereas taxa that are depleted are related to enhanced intestinal barrier integrity and reduced inflammation. While these correlations do not establish causality, they are consistent with the possibility that cold-season–associated microbial alterations may contribute to an inflammatory milieu linked to worsened ischemic injury.

### Functional analysis of gut microbiota in FMT-treated mice

Functional profiling based on KEGG ortholog (KO) annotation revealed distinct differences between the CIS-FMT and NCIS-FMT groups. β-diversity analysis of functional gene profiles using Bray–Curtis distance and PCoA demonstrated significant separation between groups (R² = 0.25, *p* = 0.013; Fig. S9). LEfSe analysis based on the KO database was conducted to compare the functional gene profiles of the gut microbiota between the CIS-FMT and NCIS-FMT groups. The results revealed that there are 43 KOs were significantly enriched and 40 KOs were significantly depleted in the CIS-FMT group. Functional annotation showed that genes associated with carbohydrate metabolism, glycolysis, fatty acid metabolism, and lipid metabolism were markedly reduced in the CIS-FMT group. Additionally, pathways related to biofilm transport, vitamin metabolism, and folate synthesis displayed a decreasing trend. Regarding amino acid metabolism, glutamate biosynthesis was upregulated, whereas lysine biosynthesis was downregulated in the CIS-FMT group (Fig. S10).

At KEGG level 2, the NCIS-FMT group showed enrichment of genes related to carbohydrate metabolism, transport, and catabolic pathways, whereas the CIS-FMT group displayed overrepresentation of genes annotated to environmental adaptation pathways (Fig. S11). At KEGG level 3, the NCIS-FMT group demonstrated higher relative abundance of genes involved in glycolysis/gluconeogenesis, ceramide metabolism, and lipoic acid metabolism. In contrast, the CIS-FMT group showed enrichment of genes mapped to KEGG pathways annotated as bacterial infection–related processes, as well as increased representation of amino acid metabolic pathways, including phenylalanine metabolism (Fig. S12).

### Metabolomic analysis of fecal samples in FMT-treated mice

Differential metabolite analysis revealed that, compared with the NCIS-FMT group, levels of palmitic acid—metabolites previously reported to be associated with pro-inflammatory responses—were significantly increased in the CIS-FMT group. In contrast, metabolites including naringenin-7-O-glucoside, propionic acid, indole-3-acetaldehyde, and fraxin were significantly reduced in the CIS-FMT group (Fig. S13). These metabolites have been reported to exhibit antioxidant, anti-inflammatory, and antiplatelet properties in various experimental contexts [28–30]. Collectively, the altered metabolite profile in CIS-FMT mice may indicate a shift toward a metabolic milieu characterized by reduced anti-inflammatory and antioxidant potential, which could be associated with more severe post-stroke injury.

### Correlation analysis of gut microbiota and metabolites in FMT-treated mice

Correlation analysis between differential metabolites and gut microbiota in FMT-treated mice was performed using Spearman correlation. Several metabolites, including cholesteryl sulfate, palmitic acid and pristimerin, displayed group-specific abundance patterns. Correlation heatmap analysis revealed significant associations between specific bacterial taxa—such as *Akkermansia muciniphila*,* Butyricimonas_virosa*,* Eubacterium_callanderi*,* Ruminococcus_G_gauvreauii*,* Clostridium_AQ_innocuum*, and *Parabacteroides_gordonii*—and these metabolites (Fig. S14). These findings indicate coordinated alterations in microbial composition and metabolite profiles in FMT-treated mice.

## Discussion

This study systematically characterized the clinical features, gut microbial profiles, and prognostic outcomes of ischemic stroke patients with cold-season onset (CIS) at the population level. Compared with non-cold season-onset patients (NCIS), the CIS group exhibited higher systemic inflammation, more severe baseline neurological deficits, and worse 3-month functional outcomes, suggesting that cold-season factors may exacerbate initial ischemic injury and impair recovery. At the microbiota level, CIS patients exhibited reduced α-diversity and distinct β-diversity patterns, indicating substantial alterations in gut microbial community structure [31, 32]. Differential analysis revealed enrichment of potentially pro-inflammatory taxa such as *Escherichia–Shigella* and *Enterococcus*, along with depletion of short-chain fatty acid-producing bacteria including *Blautia*, *Eubacterium_hallii_group*, and *Faecalibacterium*. Notably, the relative abundance of these taxa correlated with stroke severity and functional outcomes, and a predictive model incorporating five key genera demonstrated good prognostic performance (AUC = 0.848), supporting the potential of gut microbiota as a biomarker for post-stroke recovery.

Importantly, these findings demonstrate association rather than causation. The observed microbial shifts may either contribute to, or result from, systemic inflammatory changes associated with cold-season onset ischemic stroke. Given the well-established bidirectional interactions between the gut microbiota and host immune system, it is plausible that a pre-existing pro-inflammatory phenotype influences microbial composition, while microbial alterations may in turn modulate immune responses. Therefore, our human data should be interpreted as reflecting a coupled inflammatory–microbial phenotype rather than establishing a unidirectional causal pathway.

Our findings are aligned with prior clinical research reporting higher abundances of *Escherichia-Shigella* and *Enterococcus* in stroke patients with greater disease severity and poorer functional outcomes [33–35]. *Enterococcus* has been shown to stimulate pro-inflammatory pathways and is associated with adverse 90-day prognosis, while *Escherichia-Shigella* correlates with elevated inflammatory markers, supporting our observations [36–38]. In contrast, SCFA-producing bacteria such as *Blautia* and *Faecalibacterium* have been repeatedly linked to favorable neurological outcomes and anti-inflammatory effects [39–43]. Their depletion in CIS patients strengthens the hypothesis that reduced SCFA availability may contribute to increased systemic inflammation and impaired post-stroke recovery [44].

To evaluate whether season-associated microbial signatures were transmissible, we performed FMT into antibiotic-treated mice. As expected in conventional antibiotic-treated models, engraftment was partial rather than complete. Several NCIS-enriched taxa (e.g., *Ruminococcus*, *Eubacterium*) successfully colonized the murine gut, whereas some CIS-associated genera showed limited expansion, consistent with known host-specific ecological constraints, colonization resistance, and interspecies incompatibility. Importantly, post-FMT microbial profiling demonstrated persistent β-diversity separation between CIS-FMT and NCIS-FMT groups, indicating establishment of distinct microbial community structures rather than transient exposure to donor-derived metabolites. In addition, several murine-adapted genera (such as *Akkermansia*) expanded after transplantation despite not being differentially abundant in human donors, suggesting ecological restructuring within the recipient gut environment. Therefore, the FMT results reflect selective ecological transmission and functional community reorganization rather than complete taxonomic recapitulation of the donor microbiota. These findings support the biological relevance of season-associated microbial differences while acknowledging that full one-to-one transfer of human taxa is rarely achieved outside germ-free models.

In the FMT experiments, CIS-FMT mice exhibited larger infarct volumes, higher neurological deficit scores, increased circulating inflammatory markers (LPS and LBP), and reduced expression of tight junction proteins Occludin and ZO-1 compared with NCIS-FMT mice. These findings are compatible with a microbiota-associated influence on neuroinflammatory responses and intestinal barrier integrity. Similar patterns have been described in recent studies showing that gut microbial disturbances are associated with aggravated ischemic brain injury through modulation of peripheral immune activation and intestinal permeability [45–48].

Notably, CIS-FMT mice exhibited a reduced relative abundance of taxa enriched in the NCIS-FMT group, including *Akkermansia muciniphila* and *Eubacterium callanderi*, alongside enrichment of species such as *Schaedlerella arabinophila*, *Parabacteroides massiliensis*, *Streptococcus danieliae*, and *Evtetpia gabavorous*. Some of the latter taxa have been reported in association with inflammatory conditions or opportunistic pathogenic features in specific contexts, although their functional roles may vary depending on host and environmental factors. *Akkermansia muciniphila*, in particular, has been widely implicated in mucin degradation, short-chain fatty acid metabolism, and maintenance of epithelial barrier integrity [49]. Reduced abundance of *A. muciniphila* has been associated with metabolic and inflammatory disorders, and experimental supplementation has been reported to attenuate ischemic injury severity in preclinical models [50, 51]. Consistent with these observations, its relative depletion in CIS-FMT mice was accompanied by impaired intestinal barrier markers and elevated inflammatory indices. However, whether this relationship is causal or reflective of broader ecological shifts requires further mechanistic investigation [52, 53].

Further integrative analysis of fecal metagenomic and metabolomic data in FMT recipient mice revealed that, the pro-inflammatory metabolite palmitic acid was negatively correlated with several anti-inflammatory or commensal species, including *Akkermansia_muciniphila*,* Parasutterella_excrementihominis*,* Butyricimonas_virosa*,* Eubacterium_callanderi*,* Gordonibacter_urolithinfaciens*, and *Romboutsia_ilealis*. These associations suggest that cold-related gut dysbiosis may be linked to altered microbial ecology and metabolic profiles, potentially contributing to enhanced neuroinflammation and unfavorable stroke outcomes.

Our findings are consistent with accumulating evidence suggesting that environmental factors, including seasonal temperature variation, may influence host inflammatory status and gut microbial composition. Human studies have reported associations between chronic cold exposure and increased platelet activation, systemic inflammation, and alterations in gut-derived metabolites such as TMAO [12]. In independent experimental settings, some cold-associated physiological effects have been shown to be partially transferable via fecal microbiota transplantation, supporting the concept that gut microbiota can act as intermediaries between environmental stimuli and host responses. Experimental data further suggest that low ambient temperature may influence platelet signaling and reshape microbial communities through bile acid–related and metabolic pathways [54]. Within this broader framework, our results support the possibility that cold-season–associated microbial configurations are linked to an inflammatory and metabolic milieu that may exacerbate ischemic injury. However, given the observational nature of human data and the lack of direct mechanistic verification in our mouse model, a clear causal relationship cannot yet be established, and further research is needed.

## Conclusion

Our findings indicate that gut microbiota from CIS patients harbor season-associated microbial signatures that are partially transferable to recipient mice and are associated with a more pro-inflammatory gut environment. These results suggest that CIS-related dysbiosis may be linked to impaired post-stroke recovery rather than merely reflecting disease burden. Through FMT experiments, we observed that microbiota derived from CIS patients were associated with increased intestinal barrier disruption, systemic inflammation, and cerebral injury in recipient mice, potentially related to alterations in mucosal integrity and microbial metabolic profiles. Collectively, these observations are consistent with a microbiota-associated contribution to stroke outcomes. While causality cannot be definitively established within the current experimental framework, our findings provide supportive evidence that gut microbial alterations may represent a modifiable factor and a potential adjunctive target to improve ischemic stroke recovery.

### Limitations

This study has several limitations. First, the cross-sectional design allowed only single-time-point fecal sampling, limiting our ability to assess dynamic microbiome changes and their temporal relationship with clinical parameters. Second, the relatively small sample size restricted the use of more robust feature selection methods and independent external validation, thereby increasing the potential risk of model overfitting. Third, although antibiotic pretreatment was performed prior to FMT, microbial diversity in recipient mice was not systematically quantified before and after antibiotic administration. Therefore, the extent of microbiota depletion and donor engraftment could not be precisely measured. In addition, the use of antibiotic-treated rather than germ-free mice may have limited complete taxonomic transfer due to host-specific ecological constraints. Fourth, while the FMT experiments suggest an association between CIS-related microbial signatures and stroke severity, individual taxa were not functionally validated in isolation, and direct mechanistic pathways remain to be elucidated. Future studies with larger cohorts, longitudinal designs, germ-free validation models, and compositionality-aware analytical approaches are warranted to further clarify mechanistic relationships and evaluate potential causal links.

## Supplementary Information

Supplementary Material 1.

## Data Availability

The raw sequencing data generated in this study have been deposited in the NCBI Sequence Read Archive (SRA) database and are publicly available under the BioProject accession numbers PRJNA1274352 and PRJNA1275799. All sequencing runs (SRR accession numbers) can be accessed through the SRA database via the following links: (https://www.ncbi.nlm.nih.gov/sra/?term=PRJNA1274352) (https://www.ncbi.nlm.nih.gov/sra/?term=PRJNA1275799).
