# Supplementary material for: Cold environment is associated with worse outcomes in ischemic stroke patients and the underlying gut microbial mechanism
Source: BMC Microbiol. 2026 Apr 1;26:465. doi: 10.1186/s12866-026-05013-8 (PMC13181897; doi:10.1186/s12866-026-05013-8)
Supplement: Supplementary file 1 — Supplementary Material 1. [file 12866_2026_5013_MOESM1_ESM.docx]

**Supplementary materials**

**Demographics and clinical data collection**

The baseline information of all participants was collected at enrollment via direct interviews or medical records, including demographic information such as sex, age, body mass index (BMI, kg/m²), systolic blood pressure (SBP), diastolic blood pressure (DBP), medical history (hypertension, diabetes mellitus, hyperlipidemia, coronary heart disease, atrial fibrillation), smoking or drinking history, NIHSS scores upon hospitalization, and dietary habits.

Blood samples obtained after fasting were collected within 24 h of admission, which included white blood cell (WBC) counts, neutrophils, lymphocytes, monocytes, and platelets, as well as measurements of total cholesterol (TC), triglyceride (TG), high-density lipoprotein cholesterol (HDL-C), low-density lipoprotein cholesterol (LDL-C), fasting blood glucose (FBG), glycated hemoglobin (HbA1c), and homocysteine (HCY) levels. Stroke severity was evaluated by a qualified neurologist who was unaware of the study protocol, and the NIHSS score was used. The formula for calculating the neutrophil-to-lymphocyte ratio (NLR) is the absolute neutrophil count divided by the absolute lymphocyte count. The formula for the systemic immune-inflammation index (SII) is the platelet count multiplied by the NLR. The formula for the systemic inflammatory response index (SIRI) is the absolute monocyte count multiplied by the NLR.

**16S rRNA sequencing and bioinformatic analysis of human fecal samples**

Total microbial genomic DNA was extracted from fecal samples using the E.Z.N.A.® Stool DNA Kit (Omega Bio-Tek, Norcross, GA, USA). DNA quality and concentration were assessed by 1.0% agarose gel electrophoresis and a NanoDrop® ND-2000 spectrophotometer (Thermo Scientific, USA). The V3–V4 hypervariable region of the bacterial 16S rRNA gene was amplified using primers 338F (5′-ACTCCTACGGGAGGCAGCAG-3′) and 806R (5′-GGACTACHVGGGTWTCTAAT-3′). Amplicons were sequenced on the Illumina MiSeq PE300 platform (Illumina, San Diego, CA, USA). Raw paired-end reads were processed in QIIME2. Primer and adapter trimming was performed with cutadapt. Denoising and amplicon sequence variant (ASV) inference were conducted using the DADA2 plugin with default settings, and chimeric sequences were removed using the consensus-based method. Taxonomic assignment was performed using a Naive Bayes classifier trained on the SILVA 138 database trimmed to the V3–V4 region. After DADA2 denoising and chimera removal, the resulting ASV table did not contain singletons or extremely low-abundance ASVs, as these were removed during standard QIIME2 denoising and quality-filtering procedures.

To account for uneven sequencing depth, α-diversity and β-diversity analyses were performed on rarefied ASV tables (rarefied to the minimum sequencing depth across samples). β-diversity was assessed using principal coordinate analysis (PCoA) based on Bray–Curtis dissimilarity. For differential abundance analysis, non-rarefied relative abundance data were used. Group comparisons were performed using the Wilcoxon rank-sum test, and microbial biomarkers were identified using LEfSe with an LDA score > 3.5 and p < 0.05. Multiple testing was corrected using the Benjamini–Hochberg false discovery rate (FDR) method.

**Sample size estimation:**

The sample size for the FMT and MCAO experiments was determined using G*Power. Based on preliminary experimental data and effect sizes reported in previous studies, we assumed an effect size of 1.2, a power of 0.80, and an α level of 0.05. The calculation indicated that at least 6 mice per group were required. To compensate for potential losses due to antibiotic pretreatment, perioperative mortality, or unsuccessful MCAO modeling, 7 animals were included per group at the beginning of the experiment.

**Fecal microbiota transplantation (FMT)**

Five donors were randomly selected from the CIS and NCIS groups, respectively. Equal amounts of frozen fecal preparations from donors within the same group were thawed on ice and pooled to avoid repeated freeze–thaw cycles. Under anaerobic conditions, the pooled suspension was thoroughly homogenized, diluted with sterile phosphate-buffered saline (PBS), and filtered through a sterile 70 μm mesh to obtain the fecal suspension. The preparation was administered to mice immediately after filtration. All procedures were performed using sterile instruments, and separate materials were used for each group to prevent cross-contamination.

**Middle cerebral artery occlusion (MCAO)**

The MCAO model was established in mice using the intraluminal filament technique. Briefly, following anesthesia, mice were placed in a supine position on the surgical table. After shaving and disinfecting the neck area, a midline cervical incision was made to expose the left common carotid artery (CCA), internal carotid artery (ICA), and external carotid artery (ECA). The ECA was ligated and transected. A 5-0 silk suture was tied 4 mm distal to the CCA bifurcation, and a loose knot was placed around the ECA near the bifurcation using a 6-0 suture. The CCA was temporarily clamped with an arterial clip. A small puncture was made in the ECA (3 mm distal to the bifurcation), through which a silicone-coated monofilament was inserted into the ICA and advanced to occlude the origin of the middle cerebral artery (MCA). The filament and CCA were ligated, and the incision were closed in layers. During the operation, the temperature was maintained at 37 ± 0.5 °C, and the vital signs of the mice were monitored closely. After recovery, mice were allowed free access to food and water. Successful model establishment was defined by the presence of neurological deficits such as hemiparesis or body turning. Perioperative analgesia was provided via subcutaneous meloxicam (1 mg/kg).

**MCAO mouse fecal metagenomic sequencing**

Fresh fecal samples were collected from mice in the CIS-FMT and NCIS-FMT groups 24 h after MCAO, flash-frozen in liquid nitrogen, and stored at -80 °C until analysis. Total microbial DNA was extracted, and metagenomic libraries were prepared using the Illumina TruSeq DNA PCR-Free kit. Sequencing was performed on the Illumina NovaSeq platform to generate paired-end 150 bp (PE150) reads. After quality control, host DNA contamination was removed using KneadData, and high-quality microbial reads were retained.

Taxonomic profiling was conducted using MetaPhlAn3. For functional annotation, clean reads were mapped to the Kyoto Encyclopedia of Genes and Genomes (KEGG) database using kofam_scan (v1.3.0) to assign KEGG Orthologs (KOs), pathways, EC numbers, and modules. The abundance of each functional category was calculated by summing the abundances of all genes assigned to the corresponding KO, pathway, EC, or module. MetaCyc pathway profiles were obtained in parallel.

**MCAO mouse fecal metabolomics and data analysis**

Fecal samples collected from mice were placed in 1.5 mL Eppendorf tubes. Subsequently, 20 µL of internal standards—including Lyso PC17:0 (0.01 mg/mL) and L-2-chlorophenylalanine—were added to each tube. The samples were then processed using ultrasonic disruption followed by centrifugation to obtain the supernatant. The resulting supernatant was extracted using glass syringes and filtered through 0.22 μm microporous membranes. Finally, the filtrates were transferred into glass vials for subsequent analysis via liquid chromatography–mass spectrometry (LC-MS). Subsequently, the supernatants were analyzed using untargeted UHPLC-MS/MS on a Waters ACQUITY UPLC HSS T3 column coupled with a Thermo Fisher Q Exactive mass spectrometer in both positive and negative ion modes. Raw data were converted to mzXML using ProteoWizard and processed with XCMS for peak detection, alignment, and integration. Metabolites were identified by matching against HMDB, METLIN, and KEGG databases with MS/MS spectral confirmation. Data were normalized to total ion current and analyzed via PCA and PLS-DA. Differential metabolites were defined as those with projected important variable (VIP) > 1 and p < 0.05.

**Infarction volume measurement**

After deep anesthesia with isoflurane, the mice were euthanized, and their brains were harvested and sliced into 2 mm sections. Brain sections were incubated in 2% 2,3,5-triphenyltetrazolium chloride (TTC; G1017, Servicebio, Wuhan, China) at 37 °C for 15 min in the dark, then fixed in 4% PFA. Infarct area for each section was measured using ImageJ; final infarct volume (%) was calculated as (contralateral volume − ipsilateral non-infarct volume)/contralateral volume × 100 to correct for edema. Investigators performing image analysis were blinded to group identities.

**Immunohistochemical (IHC) staining** **and scoring**

Paraffin-embedded colon tissue sections were deparaffinized with xylene and rehydrated through a graded ethanol series. Antigen retrieval was performed in citrate buffer (pH 6.0). Endogenous peroxidase activity was blocked by incubation with 0.3% hydrogen peroxide, followed by blocking with 5% normal goat serum for 1 hour at room temperature. The sections were then incubated overnight at 4 °C with primary antibodies against Zonula Occludens-1 (ZO-1, 1:300, Proteintech, Wuhan, China) and Occludin (1:300, Proteintech, Wuhan, China). After rinsing with PBS, an enhancer solution was applied to cover the tissue within the hydrophobic barrier for 20 minutes. The sections were then washed twice with PBST and once with PBS. After removing excess liquid, an enhanced enzyme-labeled polymer was added and incubated for 20 minutes. Color development was achieved using DAB, and hematoxylin was used for counterstaining. Positive expression of ZO-1 and Occludin appeared as brown staining. The sections were subsequently dehydrated and coverslipped.

Images were acquired using an Olympus BX46 microscope (Olympus, Japan). Staining intensity was evaluated using Image-Pro Plus software. A semi-quantitative scoring system was used: staining intensity (none = 0, weak = 1, moderate = 2, strong = 3) was multiplied by the percentage of positively stained cells (none = 0, < 25% = 1, 25–49% = 2, 50–75% = 3, > 75% = 4) to calculate the final IHC score for each sample.


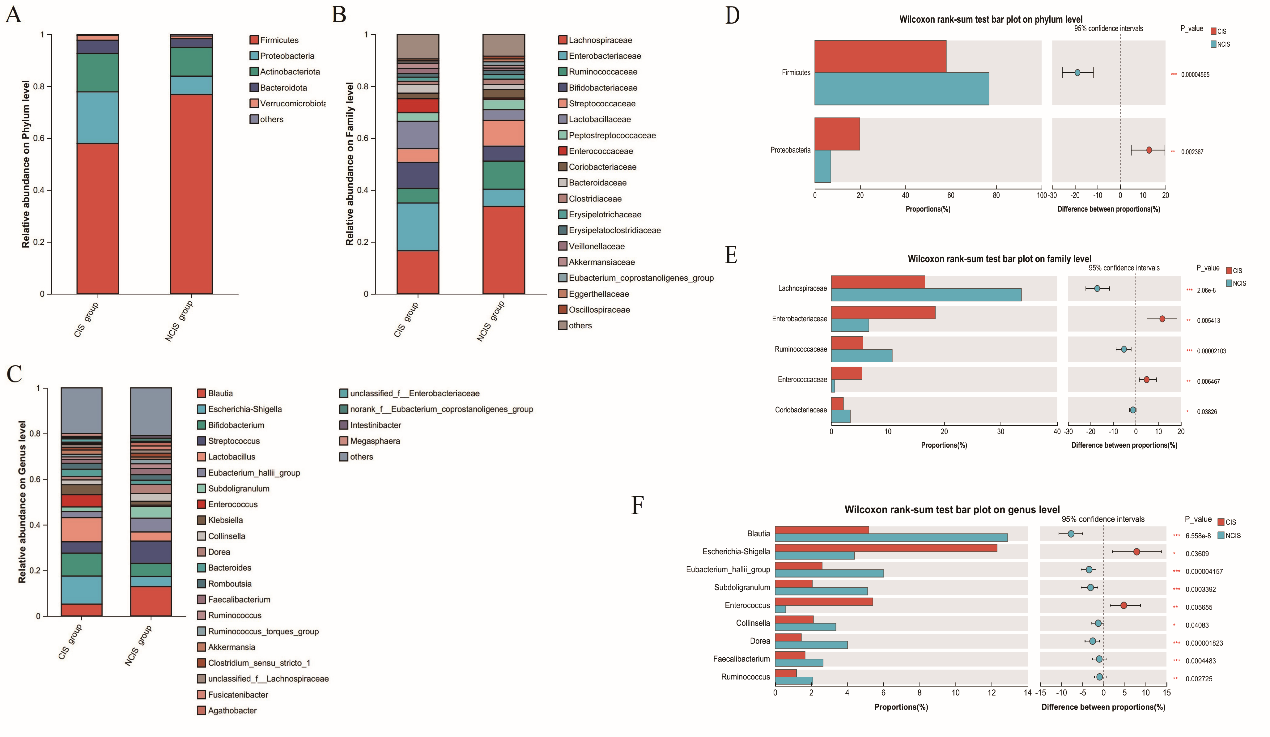


**Fig. S1 Different microbial compositions between CIS and NCIS patients.** (**A-C**) The bar plots of relative abundance at the phylum, family, and genus levels. (**D-F**) Comparison of the main categories between the two groups at the phylum, family, and genus levels. Wilcoxon rank-sum test. ^*^*p*<0.05, ^**^*p*<0.01, ^***^*p*<0.001.


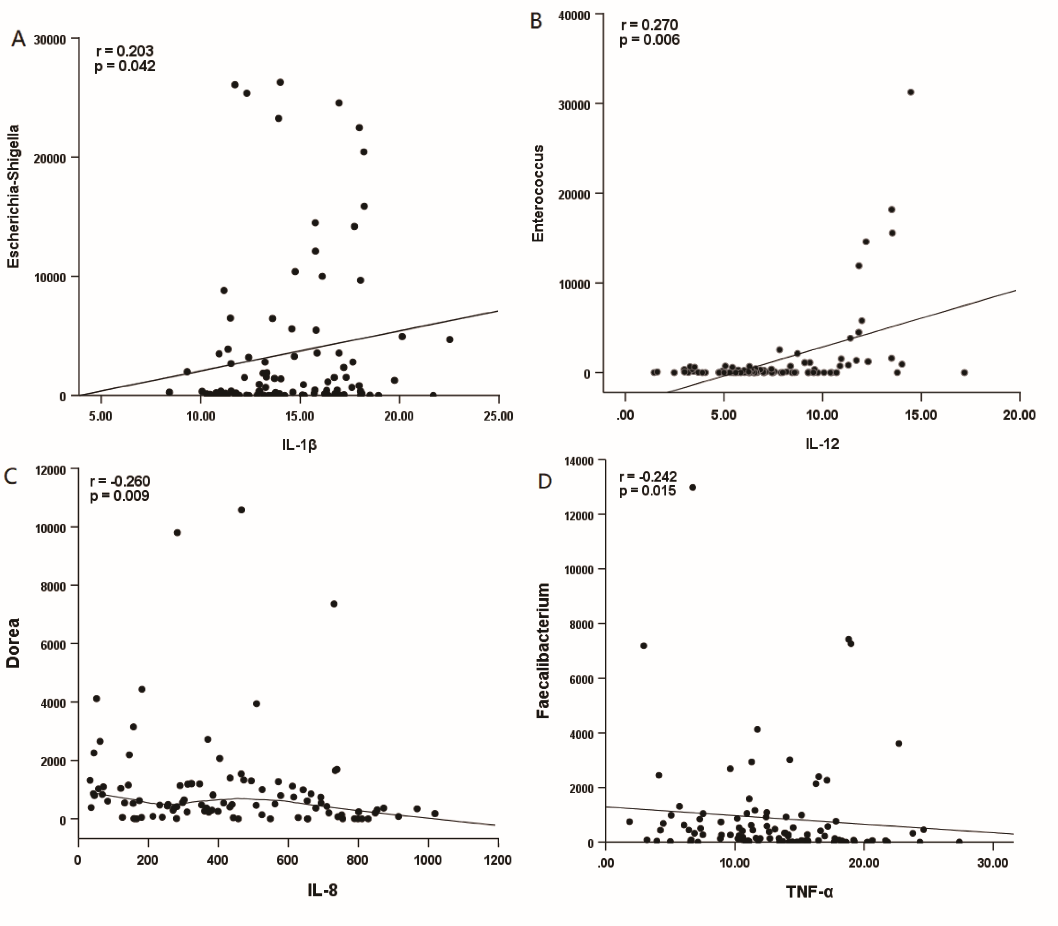


**Fig. S2 Correlations between inflammatory markers and the relative abundance of gut microbiota.** (**A**) IL-1β positively related to *Escherichia-Shigella*, (**B**) IL-12 positively related to *Enterococcus*, (**C**) IL-8 negatively related to *Dorea*, and (**D**) TNF-α negatively related to *Faecalibacterium*. *p*: probability; r: Spearman’s rank correlation.


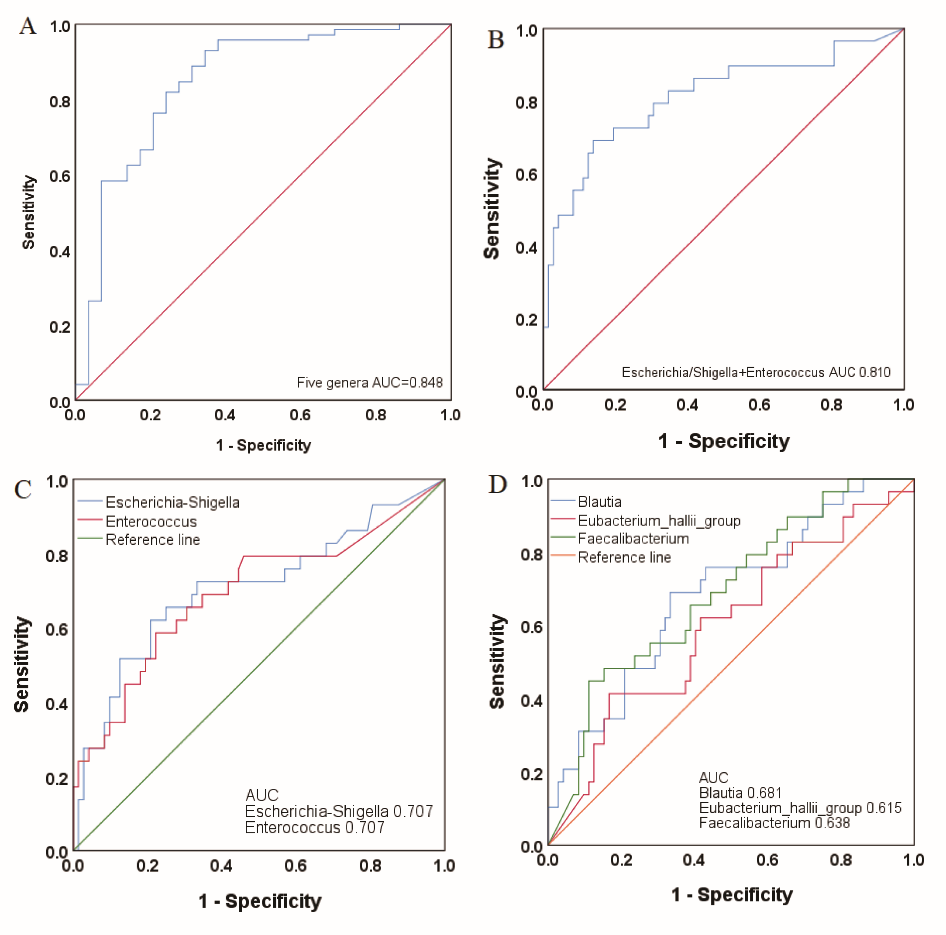


**Fig. S3 ROC curves indicating the predictive value of specific bacteria for adverse outcomes in CIS and NCIS groups.** (**A**) 5 genera combination, (**B**) *Escherichia-Shigella* and *Enterococcus* combination*,* (**C**) *Escherichia-Shigella* and *Enterococcus,* respectively, (**D**) *Blautia*, *Eubacterium_hallii_group* and *Faecalibacterium*, respectively.


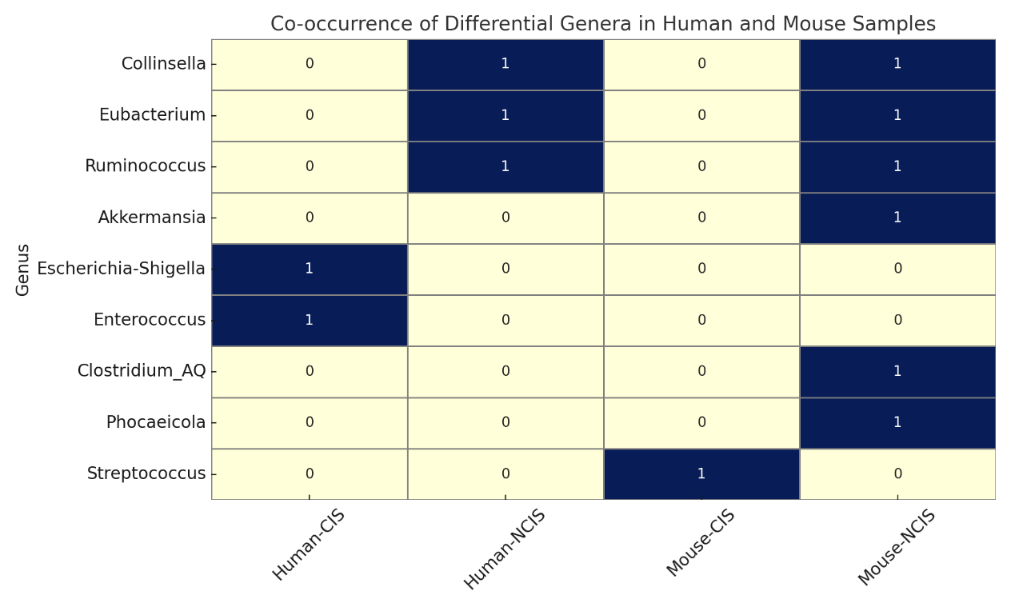


**Fig. S4 Heatmap of differentially occurring bacterial genera in humans and mice.** This figure shows the bacterial genera significantly enriched in human fecal 16S sequencing and mouse fecal metagenomics after FMT in the CIS and NCIS groups. Blue squares indicate that the bacterial genera are significantly enriched in the corresponding group (LDA > 2.5, *p* < 0.05), and yellow squares indicate that they are not enriched.


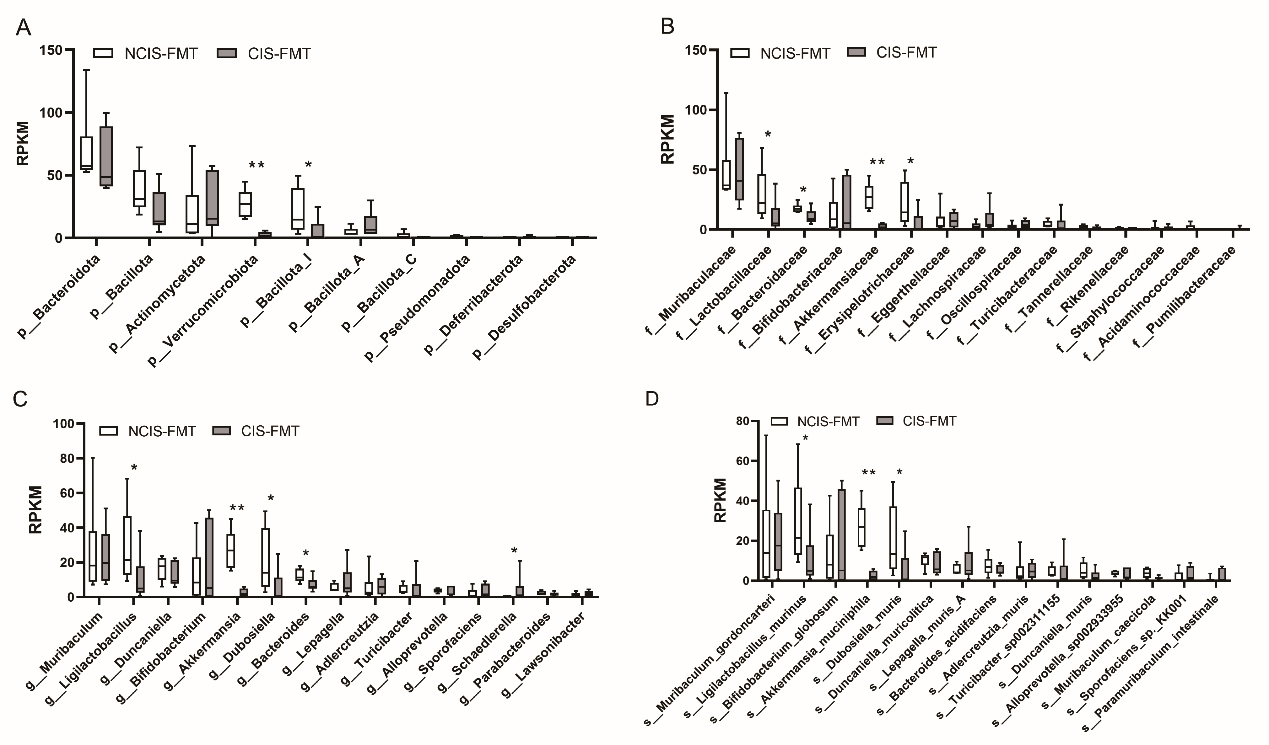


**Fig. S5 Intergroup comparison of gut microbiota at different taxonomic levels between the two groups of FMT-treated mice.** (**A**) phylum; (**B**) family; (**C**) genus; (**D**) species. Wilcoxon rank-sum test was used, ^*^*p* < 0.05; ^**^*p* < 0.01.


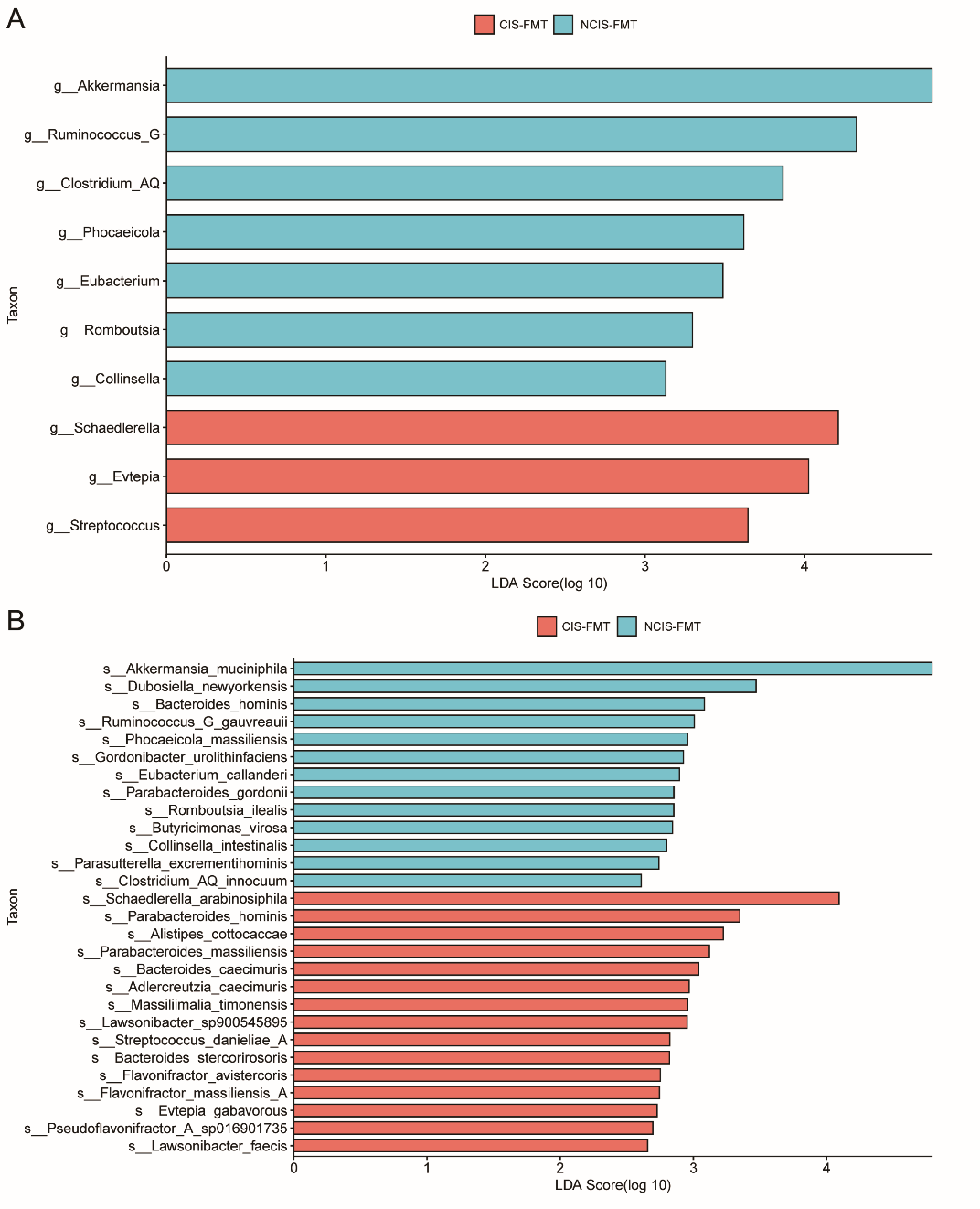


**Fig. S6 LDA bar plots comparing the gut microbiota between the two groups of FMT-treated mice.** (**A**) genus (LDA > 3); (**B**) species (LDA > 2.5).


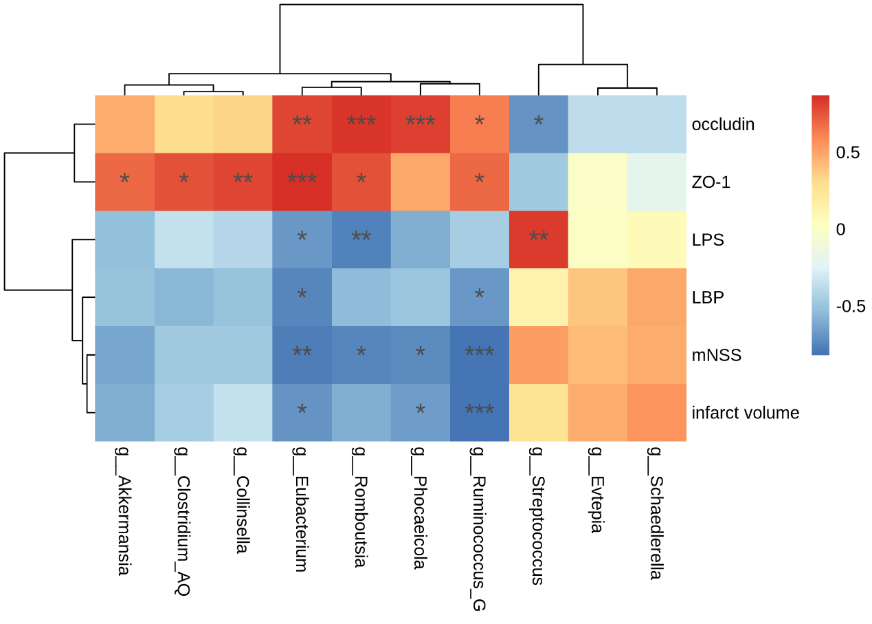


**Fig. S7 Spearman correlation heatmap showing associations between differential genera and stroke-related parameters in FMT-treated mice.** Red indicates positive correlation and blue indicates negative correlation. ^*^*p* < 0.05, ^**^*p* < 0.01, ^***^*p* < 0.001.


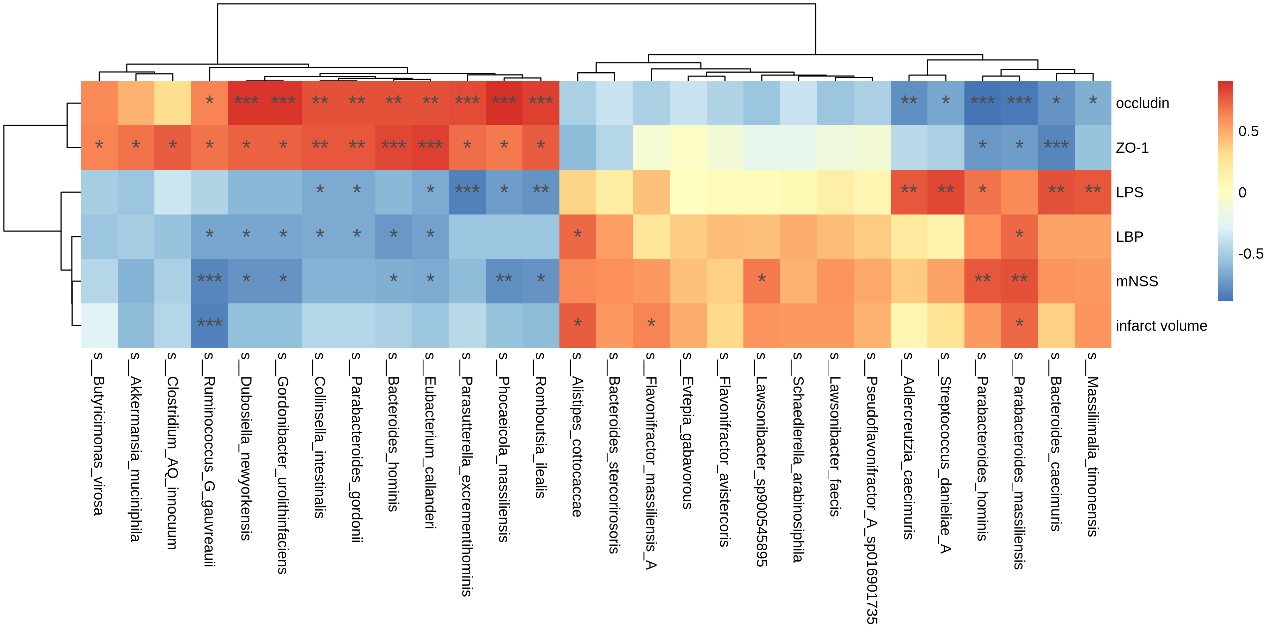


**Fig. S8 Spearman correlation heatmap showing associations between differential species and stroke-related parameters in FMT-treated mice.** Red indicates positive correlation and blue indicates negative correlation. ^*^*p* < 0.05, ^**^*p* < 0.01, ^***^*p* < 0.001.


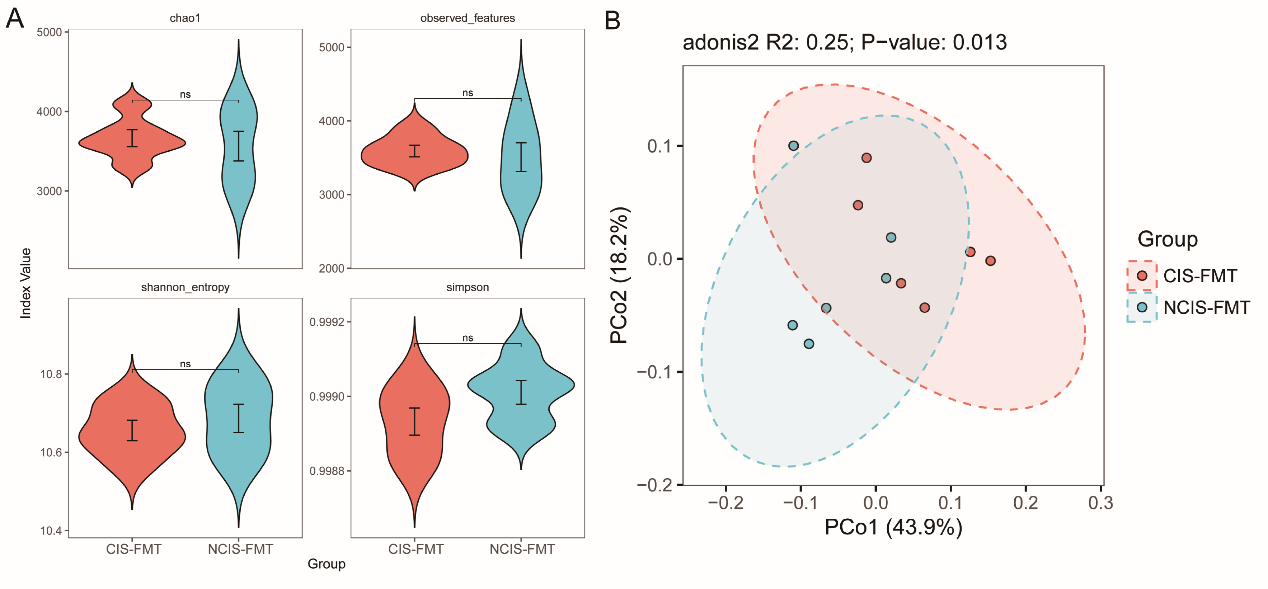


**Fig. S9 Comparison of metabolic pathway abundance diversity between the two groups of FMT-treated mice based on the KO database.** (**A**) α-diversity; (**B**) β-diversity analysis using principal coordinates (PCoA) based on the Bray-Curtis distance matrix. ns, no statistically significant difference.


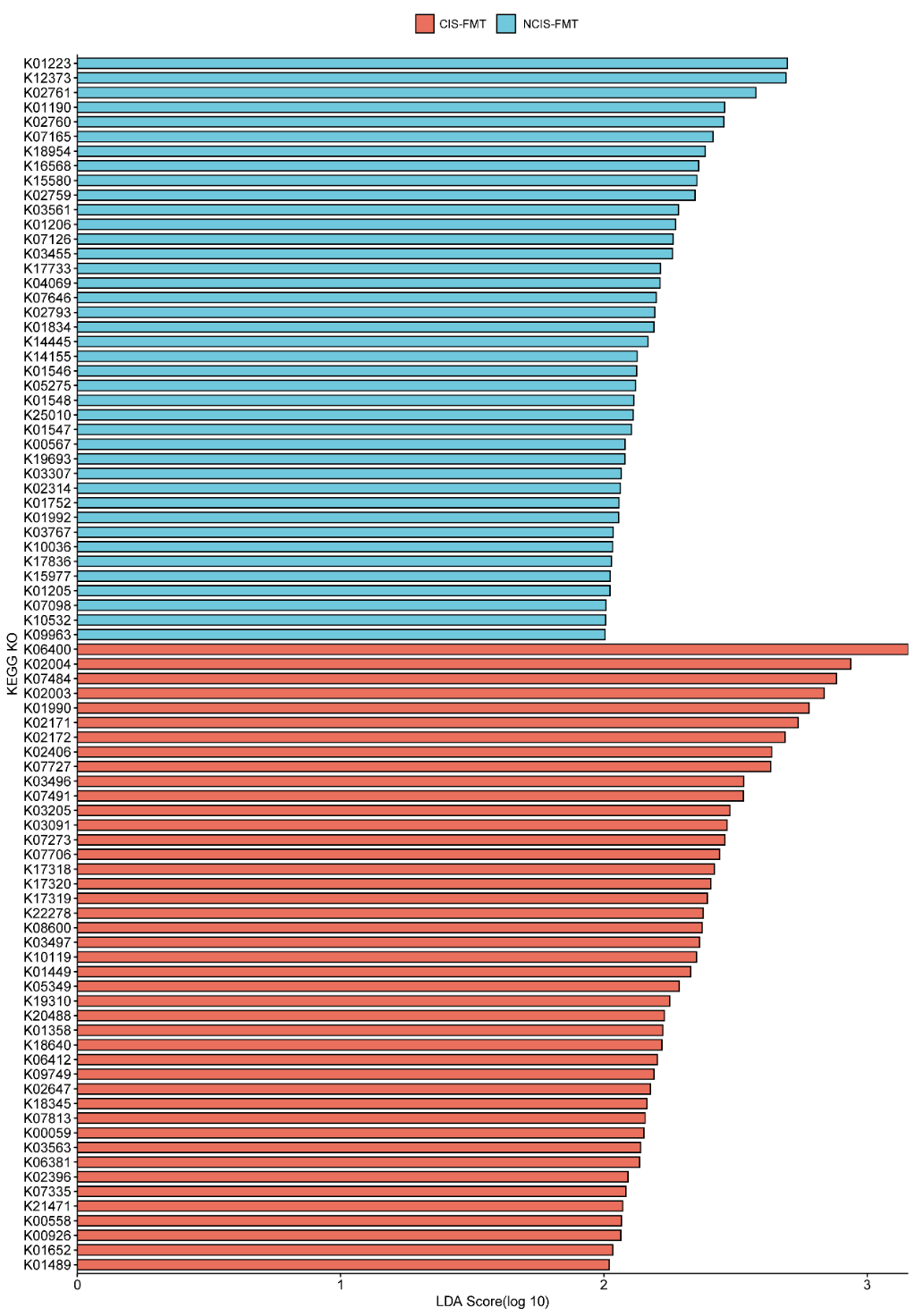


**Fig. S10 LEfSe analysis of the gut microbiota metabolic pathways between the two groups of FMT-treated mice based on the KO database (LDA > 2).**


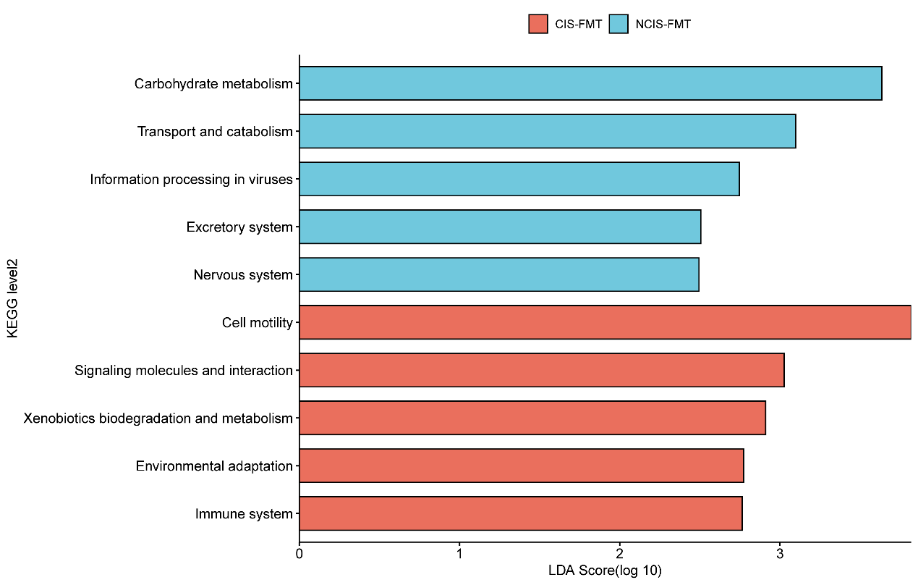


**Fig. S11 LEfSe analysis of metabolic pathways between the two groups of FMT-treated mice based on the KEGG level 2 database (LDA > 2.5).**


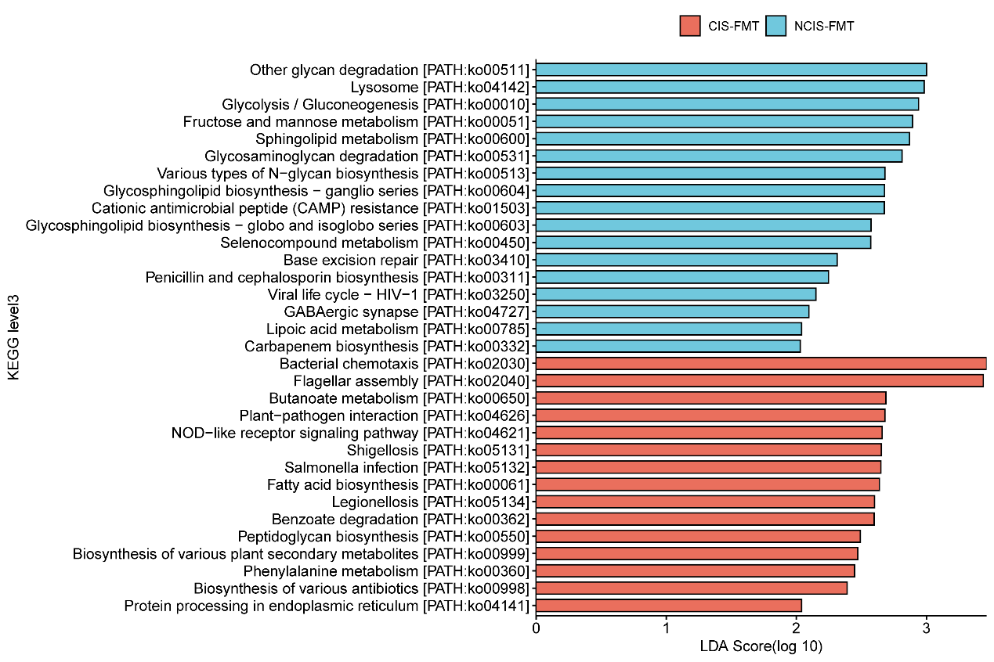


**Fig. S12 LEfSe analysis of metabolic pathways between the two groups of FMT-treated mice based on the KEGG level 3 database (LDA > 2).**


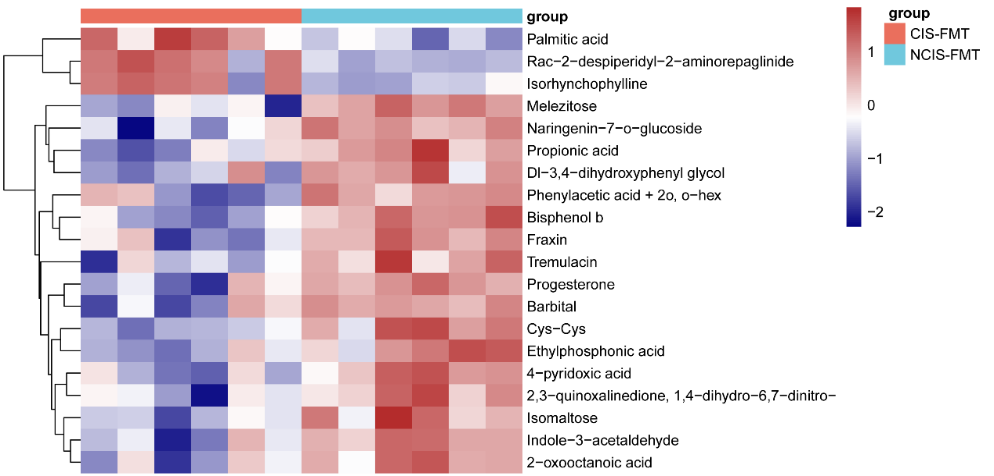


**Fig. S13 Clustering heatmap of differentially metabolites in feces of the two groups of mice after FMT.** Heatmap shows intergroup differences in metabolites of intestinal content in NEG mode in each group.


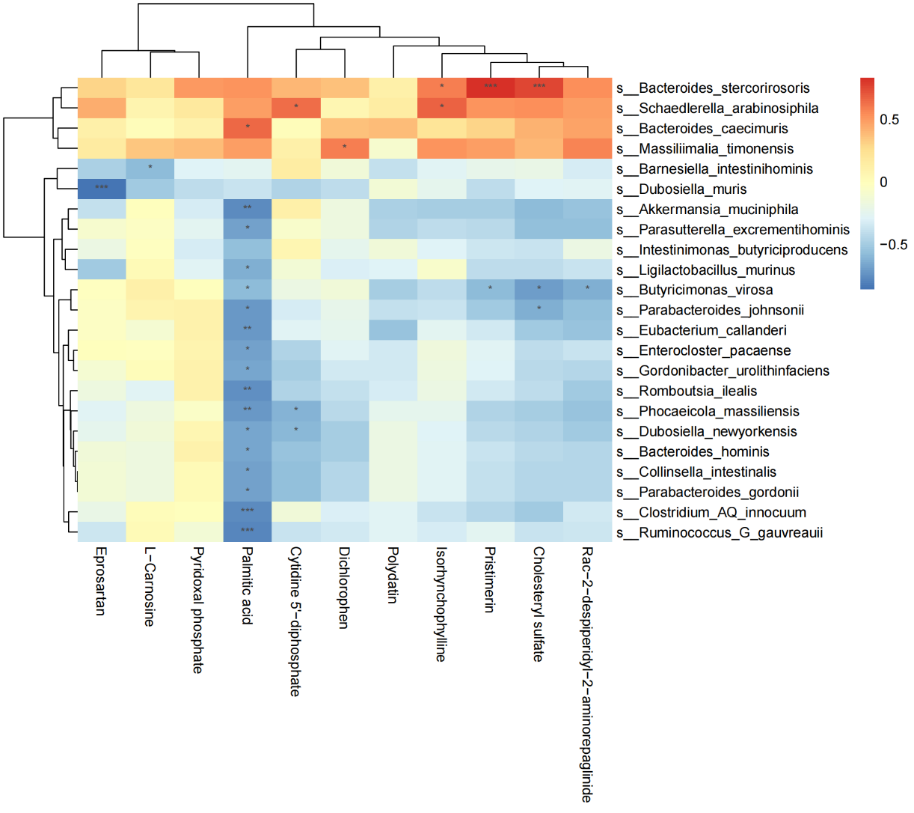


**Fig. S14 Heatmap of Spearman associations between gut microbiota and differential metabolites in FMT-treated mice.** Red indicates a positive correlation, while blue indicates a negative correlation. Data are mean ± SD. ^*^*p*＜0.05, ^**^*p*＜0.01, ^***^*p*＜0.001.

**List of abbreviations**

| Abbreviations | Definition of abbreviations |
| --- | --- |
| AIS | acute ischemic stroke |
| CIS | cold-season onset ischemic stroke |
| NCIS | non-cold-season onset ischemic stroke |
| SBP | systolic blood pressure |
| DBP | diastolic blood pressure |
| WBC | white blood cells |
| TC | total cholesterol |
| TG | triglyceride |
| HDL-C | high-density lipoprotein cholesterol |
| LDL-C | low-density lipoprotein cholesterol |
| FBG | fasting blood glucose |
| HCY | homocysteine |
| NLR | neutrophil-to-lymphocyte ratio |
| SII | systemic immune-inflammation index |
| SIRI | systemic inflammatory response index |
| BMI | body mass index |
| ROC | receiver operating characteristic |
| AUC | area under the curve |
| NIHSS | National Institutes of Health Stroke Scale |
| mRS | modified Rankin scale |
| ASVs | amplicon sequence variants |
| LDA | linear discriminant analysis |
| LEfSe | linear discriminant analysis effect size |
| FMT | fecal microbiota transplantation |
| MCAO | middle cerebral artery occlusion |
| mNSS | modified Neurological Severity Score |
| PCoA | principal coordinate analysis |
| PICRUSt2 | the Phylogenetic Investigation of Communities by Reconstruction of Unobserved States 2 |
| KEGG | Kyoto Encyclopedia of Genes and Genomes |
| IHC | Immunohistochemical |
| ZO-1 | Zonula Occludens-1 |
| TTC | 2,3,5-triphenyltetrazolium chloride |
| LPS | Lipopolysaccharide |
| LBP | Lipopolysaccharide-Binding Protein |
